# Unveiling the lead exposure attributed burden in Iran from 1990 to 2019 through the lens of the Global Burden of Disease study 2019

**DOI:** 10.1038/s41598-024-58823-z

**Published:** 2024-04-15

**Authors:** Hanie Karimi, Sara Mahdavi, Sahar Saeedi Moghaddam, Mohsen Abbasi-Kangevari, Zahra Soleimani, Zahra Esfahani, Masoud Masinaei, Sahar Mohammadi Fateh, Ali Golestani, Arezou Dilmaghani-Marand, Farzad Kompani, Negar Rezaei, Erfan Ghasemi, Bagher Larijani, Farshad Farzadfar

**Affiliations:** 1 Non-Communicable Diseases Research Center, Endocrinology and Metabolism Population Sciences Institute, Tehran University of Medical Sciences, Tehran, Iran; 2 School of Medicine, Tehran University of Medical Sciences, Tehran, Iran; 3 School of Medicine, Alborz University of Medical Sciences, Alborz, Iran; 4 Kiel Institute for the World Economy, Kiel, Germany; 5 Department of Biostatistics, University of Social Welfare and Rehabilitation Sciences, Tehran, Iran; 6 Department of Epidemiology and Biostatistics, Tehran University of Medical Sciences, Tehran, Iran; 7 Division of Hematology and Oncology, Children’s Medical Center, Pediatrics Center of Excellence, Tehran University of Medical Sciences, Tehran, Iran; 8 Endocrinology and Metabolism Research Center, Endocrinology and Metabolism Clinical Sciences Institute, Tehran University of Medical Sciences, Tehran, Iran; 9 Endocrinology and Metabolism Research Institute, Tehran University of Medical Sciences, Tehran, Iran

**Keywords:** Environmental pollutants, Cardiovascular diseases, Death, Disability-adjusted life years, Global burden of disease, Lead, Environmental sciences, Natural hazards, Cardiology, Nephrology, Neurology, Risk factors

## Abstract

This study aimed to investigate the estimated burden attributed to lead exposure (LE), at the national and subnational levels from 1990 to 2019 in Iran. The burden attributed to LE was determined through the estimation of deaths, disability-adjusted life years (DALYs), years of life lost (YLLs) and years lived with disability (YLDs) using the comparative risk assessment method of Global Burden of Disease (GBD) study presenting as age-standardized per 100,000 person year (PY) with 95% uncertainty intervals (95% UI). Furthermore, the burden of each disease were recorded independently. Eventually, the age-standardized YLLs, DALYs, deaths and YLDs rates attributed to LE demonstrated a decrease of 50.7%, 48.9%, 38.0%, and 36.4%, respectively, from 1990 to 2019. The most important causes of LE burden are divided into two acute and chronic categories: acute, mainly causes mental disorders (DALYs rate of 36.0 in 2019), and chronic, results in cardiovascular diseases (CVDs) (DALYs rate of 391.8) and chronic kidney diseases (CKDs) (DALYs rate of 26.6), with CVDs bearing the most significant burden. At the sub-national level, a decrease in burden was evident in most provinces; moreover, low and low-middle SDI provinces born the highest burden. The burden increased mainly by ageing and was higher in males than females. It was concluded that although the overall decrease in the burden; still it is high, especially in low and low-middle SDI provinces, in advanced ages and in males. Among IDID, CKDs and CVDs that are the most important causes of LE-attributed burden in Iran; CVDs bear the highest burden.

## Introduction

GBD 2019 has incorporated various environmental risk factors including four groups of unsafe water and sanitation, air pollution, non-optimal temperature, and other environmental risk factors. Residential radon and lead exposure (LE) belong to the fourth group^1^.

Lead (Pb) is a hazardous heavy metal environmental toxin with many harmful health effects. While lead is a toxic metal, its characteristics have made it widely used in various industries, leading to environmental contamination and various long-term impacts on human health^2^. Two main sources of lead encompassing natural and synthetic sources are known exist. Lead is mainly found in the air, soil, water and different food products, as well as various synthetic sources^3^.

Historically, lead has been extensively used in industry and various products, such as petroleum, batteries, paints, and pipes^4^. Inhalation and gastrointestinal entry are the most prevalent routes of lead entry into the body. In adults, lead absorption from inhalation is estimated to range between 20 and 60%. Although gastrointestinal absorption rate is lower in adults, around 10%, the ratio could be as high as 50% in children^5^. Lead exposure poses a severe risk to human health due to its cumulative effect and non-biodegradable nature^6^. The absorbed lead can be stored in soft tissues, such as the kidney, central nervous system (CNS), and bones^7^. LE is a risk factor for non-communicable diseases (NCDs), such as cardiovascular diseases (CVDs) and chronic kidney diseases (CKDs), and developmental cognitive damage like idiopathic developmental intellectual disability (IDID)) at younger ages, even in small doses^8–11^; which could have a significant burden on public health in terms of disability or mortality^12^. Nevertheless, no safe level for LE has been established yet^13^. Moreover, NCDs have become a significant public health issue in developed and developing countries^14^. LE resulted in 900,000 deaths and 21,700,000 DALYs in 2019, assigning a substantial disease burden^2^. The exposure to lead in Iran can occur through leaded gasoline, although prohibited, and industrial LE with the potential for contaminating air, water and soil. Besides, several foods originating from the contaminated environment, such as fish, rice, tea, vegetables, raw food, milk, and bread might also be affected. It is reported that the plant food lead contamination is higher than allowable limits in most studies conducted in Iran which can result in several health problems^15,16^.

Furthermore, the prevalence of lead in several drugs, particularly herbal ones, opium, children's toys and cosmetics, has increased its exposure^16^. Historically, LE has been used in various industrial settings and products, such as cosmetics, paints, petroleum, and pipes, and is one of the earliest known causes of occupational disease^17,18^. Although evidence was present regarding the pathological aspects of contact with LE, it was not until the late twentieth century that legislation and regulations were implemented to reduce LE, which caused a reduction in associated diseases^18^. Occupational and environmental legislation aimed at decreasing LE has also been passed in Iran^16^. Due to the regulations made to reduce the LE in Iran and all over the world, from 1990 to 2019, the amount of LE has decreased by about 1% per year globally^12^. Nevertheless, the exposure remains high, as half of the 2,000,000 lives lost to chemical exposure in 2019 were attributed to LE^2^.

Thus, the progress towards reducing the burden attributable to LE needs to be investigated to help determine whether previous efforts have been adequate and assist policymakers in developing better regulatory rules and making evidence-based decisions.

The current study aims to discuss the burden attributed to LE and compare attributable death, years of life lost (YLLs), years lived with disability (YLDs) and disability-adjusted life years (DALYs) of different diseases induced by LE according to the results of GBD 2019, among 31 provinces of Iran from 1990 to 2019.

## Material and methods

### Overview and data resources

The data was obtained from the GBD 2019, available at the Institute for Health Metrics and Evaluation (IHME) website (http://ghdx.healthdata.org/gbd-results-tool), in which to determine risk exposure for each risk factor, published studies and systematic reviews, surveys, censuses, reports, cross-sectional studies, ground monitor data, and administrative data in Iran have been evaluated from 1990 to 2019.

### Comparative risk assessment method

Since GBD 2002, the attributable burden for each risk factor has been based on comparative risk assessment (CRA). The following analytical steps are included in the CRA: (1) risk-outcome pair selection, (2) relative risk estimation as an exposure function according to systematic reviews or at least two cohort studies and running a meta-analysis of the relative risks (for new risk-outcome pairs, after excluding potential bias and confounding, in the condition of P-value < 0.05 the association is proven to be statistically significant). (3) Estimation of the level of exposure in each age-sex-location-year by spatiotemporal Gaussian process regression (ST-GPR) or DisMod-MR 2.1 (Bayesian statistical models) (4) determination of the counterfactual level of exposure called theoretical minimum risk exposure level (TMREL) (5) computation of population attributable fraction (PAF) and calculating attributable death, YLLs, YLDs and DALYs by multiplying PAF by the rates in each age–sex–location–year groups. (6) Estimation of PAF and attributable burden for risk factor combinations^12^.

### Lead exposure-related diseases

According to the GBD 2019 study, LE-related diseases are classified into three types: CVDs, CKDs and mental disorders. CVDs related to LE include endocarditis, peripheral artery disease, aortic aneurysm, atrial fibrillation and flutter, cardiomyopathy and myocarditis, non-rheumatic valvular heart disease, hypertensive heart disease, stroke (subarachnoid hemorrhage, intracerebral hemorrhage and ischemic stroke), ischemic heart disease (IHD), rheumatic heart disease (RHD) and other cardiovascular and circulatory diseases. As a CVDs subtype, stroke has three subtypes: subarachnoid hemorrhage, intracerebral hemorrhage, and ischemic stroke. Subclasses of diseases in CKDs that have been surveyed include chronic kidney disease due to the following causes: diabetes mellitus type 1, diabetes mellitus type 2, hypertension, glomerulonephritis, and chronic kidney disease due to other and unspecified causes. Mental disorders have one subtype related to LE: IDID.

### Attributed burden indices

The attributed burden of acute and chronic LE in Iran is demonstrated with YLLs, YLDs, DALYs (the sum of YLLs and YLDs) and death in two types of all ages and age-standardized rate per 100,000 population in both sexes at national and subnational levels with 95% uncertainty intervals (95% UI)^12^.

### Socio-demographic index

The socio-demographic index (SDI) is used in GBD studies to measure sociodemographic development based on per capita income, educational income, and total fertility rate. It is classified into five quintiles: high, high middle, middle, low middle and low. The current study compares the burden attributed to LE in different SDI quintiles.

### Statistical analysis

Data on the LE was gathered from published literature and surveys conducted in Iran, which was extracted from the global health data exchange system (GHDx)^1^. Blood lead level (BLL) and bone lead levels were extracted from studies that take and analyze blood samples. By lifetime estimated blood lead, cumulative blood lead index can be obtained for estimating bone lead levels.

Blood lead exposures are reported in arithmetic mean, geometric mean or median forms. As in GBD 2019, MR-BRT adjustment variables are utilized to modify all blood LE levels in the arithmetic mean form (reference form) to make comparisons easier. The Bayesian statistical model in this study is the ST-GPR, which is used for exposure modelling, e.g., showing the blood LE distribution as a curve for each year of the lifetime and using the area under this curve (cumulative blood lead index) to determine bone lead; or, when there is insufficient data about a place or a time, covariates related to that time or place can be used by ST-GPR modelling. In conclusion, the ST-GPR is an estimating tool for predicting BLL means and standard deviations in all GBD locations, all ages, and sexes during any given period.

Available evidence declares that BLL, used for acute LE, is related to the IDID; the relative risks of this association in different BLLs are adapted from a 2013 paper^19^; and bone lead level, used for chronic LE, is related to a rise in systolic blood pressure and eventually to its cardiovascular and renal outcomes. The relative risks for bone LE and related diseases are adapted from a 2008 meta-analysis^20^.

In the current study, the cut-off value for LE is based on the TMREL of lead, adapted from GBD 2019, which is considered 2.0 μg/dL; however, in some regions, it might be much higher or lower (e.g., the USA which has had a TMREL of 1.2 μg/dL for BLL since 2009–2010^21^).

Finally, YLLs, YLDs, DALYs and deaths were used to calculate the burden of diseases at national and subnational levels. Data were presented as age-standardized per 100,000 person year (PY) with 95% uncertainty intervals (95% UI). Besides, the calculation of percent change was based on the beginning (1990) and ending (2019) years of the mentioned study period. Moreover, the attributed burden was compared in different age groups, for both sexes and in various SDI quintiles. The visualization and analysis of the data and depiction of Figures were performed by STATA v.13.1 and RStudio v 1.4.1106.

## Results

The all-cause and cause-specific burdens attributed to LE measured with deaths, DALYs, YLLs and YLDs are described in the following sections at the national and subnational levels in Iran. The LE cause-specific burdens are divided into two acute and chronic causes and their subcategories’ burden are discussed furtherly.

### All-cause burden

**Figure 1:**
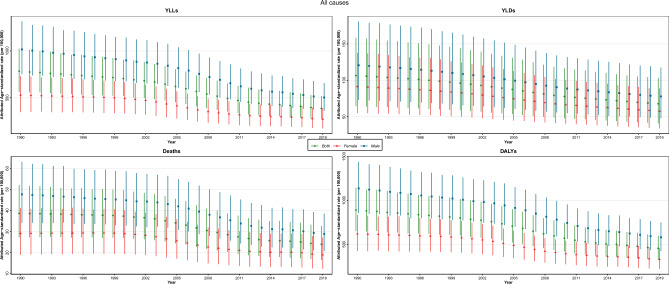
Time trend of age-standardized attributed burden rate to lead exposure by sex in Iran, 1990–2019.

**Figure 2:**
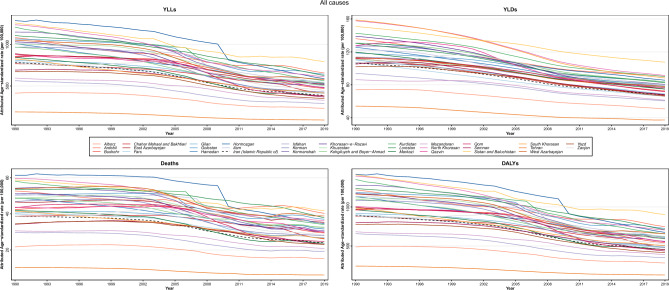
Time trend of age-standardized attributed burden rate to lead exposure at national and provincial, 1990–2019.

From 1990 to 2019, a nearly declining trend in all-cause deaths, DALYs, YLLs, and YLDs rates attributed to LE was seen for both sexes (Fig. 1). There was a divergence between provinces for deaths, DALYs, YLLs and YLDs attributed to LE in 1990, which got more convergent in 2019 (Fig. 2).

The age-standardized deaths rate (ASDR) per 100,000 decreased by 38.0% (95% UI 45.5–31.5) from 38.7 (27.3–52.1) in 1990 to 24.0 (16.5–32.7) in 2019. Similarly, the changes of DALYs, YLLs and YLDs in the same time interval showed a declining trend as the following: the age-standardized DALYs rate reached 454.4 (323.2–597.6) per 100,000 in 2019, indicating a − 48.9% (− 54.6 to − 44.3) decrease. The highest rate change was related to YLLs with a − 50.7% (− 56.9 to − 45.7) change; however, age-standardized YLDs rate had the lowest change in the mentioned time interval (− 36.4% (− 41.6 to − 31.0)).

The ASDR attributed to LE among 31 provinces of Iran varied from 6.1 (3.3–9.4) in Tehran to 41.8 (30.5–55.6) in Sistan and Baluchistan; Hormozgan with the highest death rate in 1990, reached 34.5 in 2019, which was nearly half of 1990. The age-standardized DALYs rate ranged from 127.8 (71.8–192.2) per 100,000 to 901.4 (673.8–1167.2) in 2019, with the same pattern for deaths: the highest and lowest provinces almost halved in 2019 compared to 1990. In terms of the age-standardized YLLs, in 2019, it ranged from as low as 90.5 (45.5–143) per 100,000 to as high as 793.9 (574.2–148.9), with a 50% decrease in the lowest and highest rates of 1990. According to the age-standardized YLDs rate in 2019, the lowest and highest were 37.3 (20.1–60.7) per 100,000 and 107.5 (68.2–156.6). Tehran and Sistan and Baluchistan were the two provinces with the lowest and the highest rates, respectively for all mentioned rates.

### CVDs burden

CVDs attributed to LE accounted for an ASDR of 22.6 (15.4–31.1) in 2019, indicating a decrease (− 39.2% (− 46.6 to − 32.5)) since 1990. Similarly, the age-standardized DALYs, YLLs and YLDs revealed decreasing trends at the same period as follows; a − 50.3% (− 56.6 to − 45.3), − 51.6% (− 57.9 to − 46.5) and − 23.5% (− 30.4 to − 18.1) reduction to 391.8 (270.8–528.6), 365.5 (253.8–494.5) and 26.3 (16.1–40.0) in 2019 was reported for the changes of DALYs, YLLs and YLDs, respectively.

Among subnational provinces, the ASDR varied from 9.5 (14.9–15.9) in Tehran to 58.5 (39.6–79.8) in Hormozgan and 5.6 (2.9–8.8) in Tehran to 37.7 (27–51.1) in Ardebil in 1990 and 2019, respectively, showing a decreasing trend over time. This trend coincided, even for the DALYs, YLLs, and YLDs. Their reduced 2019 rates are as follows: DALYs spanned from 95 (47.3–149.7) to 755.6 (548.6–1000.2), YLLs from 83 (41.3–132) to 712.4 (511.6–950.4) and the YLDs from 12.0 (5.5–20.5) to 43.2 (28.6–61.9); With Tehran being the province with the lowest rate and Sistan and Baluchistan with the highest rate for all. (Supplementary Fig. 1).

Top three CVD subtypes with the highest national burden, based on DALYs rate, in 2019 are further discussed in the following (from highest to lowest) at national level, the data regarding their subnational burden is demonstrated in Supplementary File.

#### Ischemic heart disease

IHD bears the highest burden among CVDs subtypes. The most significant changes were for YLLs and DALYs with − 55.4% (− 61.8 to − 50.0) and − 54.8% (− 61.1 to − 49.4) downturn to 211.9 (146.5–290.4) and 217.9 (150.7–298.4) in 2019, respectively. After that, the ASDR showed a change of − 43.4% (− 50.8 to − 36.6) over this period to 12.9 (8.6–18.1); and YLDs reached 6.0 (3.5–9.6) at the end of this time interval indicating a − 10.6% (− 18.6 to − 4.0) diminution.

#### Stroke

The second subtype with the highest burden after IHD is stroke. The pattern of stroke rates at the national level was declining as well. The death rate decreased to 4.6 (3.0–6.4) in 2019, which showed a − 45.8% (− 54.0 to − 35.7) reduction compared with 1990. Besides, DALYs rate decreased by − 53.2% (− 60.6 to − 45.7) from 1990 to 87.5 (57.8–117.9) in 2019; YLLs rate showed a reduction almost similar to DALYs with − 55.5% (− 62.8 to − 47.6) change from 1990 to 75.4 (50.1–101.3) in 2019. The least change was for YLDs, which demonstrated a − 31.0% (− 38.8 to − 24.8) reduction over 30 years to 12.0 (7.0–18.3) in 2019.

The burden attributed to 3 stroke subtypes, including the subarachnoid hemorrhage, intracerebral hemorrhage and ischemic stroke is further described in the Supplementary File.

#### Hypertensive heart disease

All burden measure rates had a declining pattern at the national level during the time interval from 1990 to 2019. Deaths rate with − 13.9% (− 40.4 to 10.3), DALYs rate with − 28.0% (− 48.9 to − 8.9), YLLs with − 28.8% (− 50.4 to − 8.7) and YLDs with − 12.3% (− 26.9 to − 2.0) reduction reached to the following rates in 2019, respectively: 4.3 (1.4–9.4), 68.8 (28.5–137.5), 64.4 (26.4–129.1) and 4.4 (1.7–9.3).

### CKDs burden

Investigating the burden attributed to diabetics and CKDs due to LE over 30 years demonstrated a similar downward trend: decreased age-standardized deaths, DALYs, YLLs, and YLDs rates. The ASDR, with a change of − 11.1% (− 28.2 to − 0.95) reached 1.4 (1.0–1.8) in 2019. The DALYs rate changed from − 23.3% (− 32.7 to − 0.16) to 26.6 (18.3–35.9) in 2019. Meanwhile, the YLLs rate demonstrated a similar trend and reached 21.2 (14.9–28.2) (with a percent change of − 27.1% (− 37.7 to − 19.1)), and YLDs change was slightly intangible compared to others: − 4.1% (− 14.3 to − 0.7) to 5.4 (3.2–8.4) in 2019.

Analysis of the burden at the subnational level revealed the following outcomes: the lowest and highest ASDR in 2019 was 0.6 (0.3–0.8) (Tehran) and 4.6 (3.3–6.1) (Sistan and Baluchistan), respectively; unlike other trends, the ASDR due to CKDs showed an increasing trend in some provinces like top four provinces with the highest ASDR in 2019: 1. Sistan and Baluchistan (4.1–4.6) 2. Ilam (from 2.5 to 3.1) 3. Ardebil (from 2.1 to 2.6) 4. East Azerbaijan (from 2.3 to 2.5) (Supplementary Fig. 2). This increasing trend in some provinces was also evident regarding the age-standardized DALYs, YLLs and YLDs due to CKDs. DALYs rate spanned from 10.0 (5.2–15.7) to 92.5 (66.3–121.2) in 2019. Regarding the YLLs rate, the range was from 7.5 (3.9–11.7) to 81.5 (58.1–107.7). Furthermore, the YLDs rate revealed the lowest and the highest rates of 2.6 (1.2–4.6) and 11 (6.9–16.3) in 2019; Noting that Tehran was the province with the lowest rate and Sistan and Baluchistan was the area with the highest rate for DALYs, YLLS and YLDs.

### IDID burden

Acute LE causes mental disorders at an early age, and the only type involved in LE is IDID. National, the DALYs rate decreased by − 45.8% (− 54.2 to − 40.3) to 36.0 (15.3–65.3) in 2019. Among subnational provinces of Iran, the lowest to highest DALYs rate reached 22.8 (8.3–43.3) (Tehran) to 53.3 (24.5–93) (Sistan and Baluchistan) in 2019. In terms of YLDs in 2019, the total range was from 22.8 (8.3–43.3) (Tehran) to 53.3 (24.5–93) (Sistan and Baluchistan), representing a significant decrease since 2019 (Supplementary Fig. 3).

### Attributed burden by SDI regions

**Figure 3:**
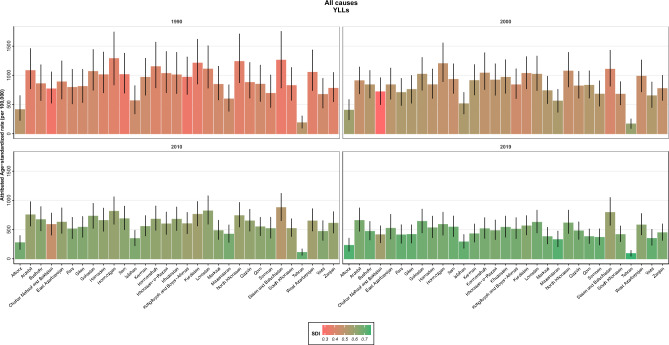
Provincial age-standardized attributed YLLs rate to lead exposure by SDI in 1990, 2000, 2010 and 2019. YLLs: Years of Life Lost; SDI: Socio-Demographic Index.

At the SDI level, the general trend was as follows: low and low-middle provinces represented the highest rates of age-standardized deaths, DALYs, YLLs and YLDs rates due to all causes in both 1990 and 2019. On the other hand, provinces in the high SDI quintile acquired the lowest rates in both years. A closer look at the rates revealed that the highest death rates were for low-middle and low SDI regions in 1990 and 2019. In contrast, high SDI provinces revealed the lowest death rates in both years. Regarding the DALYs rate, the highest rate was seen in low and low-middle SDI regions in 1990 and the low SDI region in 2019, while the lowest rate was reported in a high SDI province in both years. YLDs rate had the same pattern as DALYs; YLLs rate's lowest rate was the same as others in high SDI regions in both years. Concerning the highest YLLs rates, low-middle and low-SDI provinces demonstrated this feature in 1990 and 2019, respectively. The critical point comparing 1990 and 2019 is the reduction of almost all rates from 1990 to 2019 in all SDI quintiles (Fig. 3). Another noteworthy point is the improvement of SDI from 1990 to 2019, accompanying a reduction in all causes burden attributed to LE (Supplementary Figs. 4–7).

**Figure 4:**
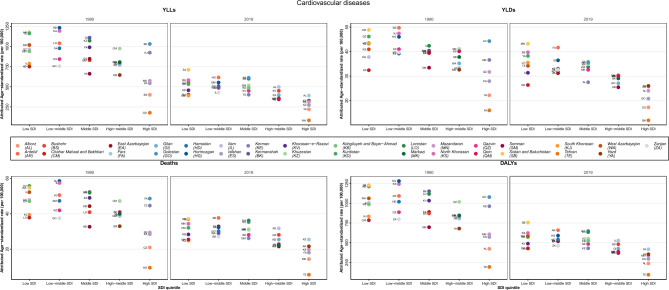
Distribution of provincial age-standardized burden rate due to cardiovascular diseases attributed to lead exposure by SDI quintiles, 1990 and 2019. SDI: Socio-Demographic Index.

CVDs caused by LE revealed a relatively similar pattern to all-cause burden in terms of SDI; however, some subtypes, including peripheral artery disease and atrial fibrillation and flutter, revealed an increasing pattern among their deaths, YLLs and YLDs (in peripheral artery disease) rates from 1990 to 2019 (Fig. 4, Supplementary Figs. 8 and 9). Moreover, some other subtypes demonstrated different relations between the minimum and maximum rates and SDI as follows: some middle SDI provinces had the highest burden of cardiomyopathy and myocarditis and subarachnoid hemorrhage; or non-rheumatic valvular heart disease had the highest burden in high-middle SDI provinces, especially in 1990 and the lowest rate of YLDs in a low SDI province in 2019, unlike the general trend. Additionally, ischemic stroke is another example that had the highest rates of YLLs, YLDs and DALYs in high SDI provinces in 1990. Besides, RHD revealed the lowest rate of YLDs in middle and high-middle SDI provinces (Supplementary Figs. 10–14).

**Figure 5:**
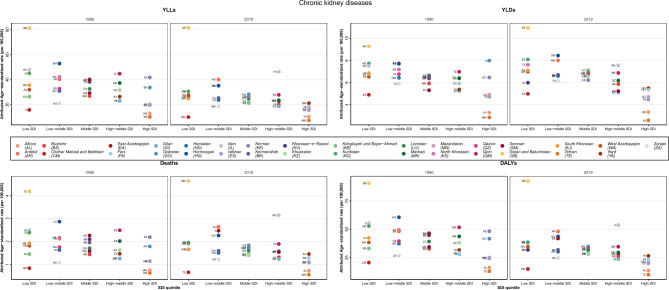
Distribution of provincial age-standardized burden rate due to chronic kidney diseases attributed to lead exposure by SDI quintiles, 1990 and 2019. SDI: Socio-Demographic Index.

CKDs had an almost identical pattern of deaths, DALYs, YLLs and YLDs rates to all causes burden by SDI level at the same period (Fig. 5).

### Attributed burden by age and sex distribution

**Figure 6:**
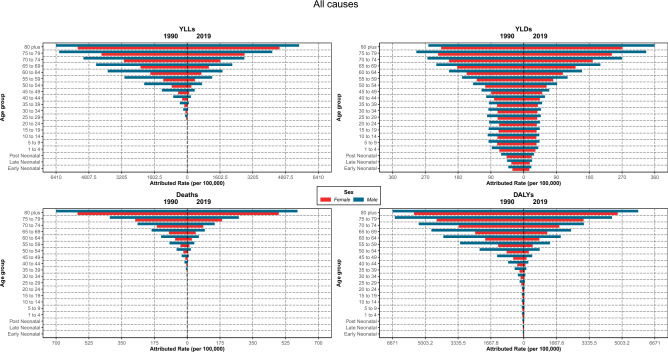
Attributed burden rate to lead exposure by age groups and sex in Iran, 1990 vs 2019.

The ASDR, DALYs and YLLs rates attributed to LE had similar age patterns, all rates increased with aging, and the rates in 2019 were lower compared to the same age group in 1990; moreover, comparing the rates between males and females in each age group, we found that males had significantly higher rates than females, illustrating a disparity in care among sexes. Regarding the YLDs rate, there was an increasing trend by aging; however, in the age group between 5 and 39 years, the rate was almost constant in 1990 and 2019. Furthermore, in all age groups, the YLDs rates were higher in 1990 compared to 2019 in both sexes, but in the age group of 70 plus, the rates were higher in 2019 than in 1990 in males, and this pattern was evident in females 75 years and older. The pattern of higher rates in males than females was obvious in the YLDs rates as well (Fig. 6).

**Figure 7:**
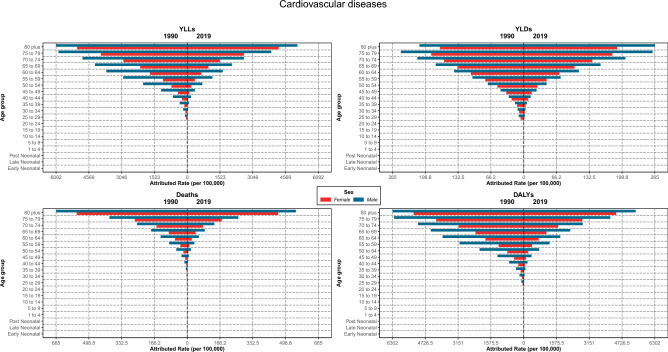
Burden rate due to cardiovascular diseases attributed to lead exposure by age groups and sex in Iran, 1990 vs 2019.

Regarding the CVDs burden, all rates increased by aging in both genders in both years (1990 and 2019), and it was always higher in males compared to females; moreover, the rates of each age group in 1990 was significantly higher than the same age group in 2019 for both genders. YLDs rate followed a slightly distinct pattern; it increased with aging in both genders; however, in 1990, the rate of the 80 plus age group was lower than 70–74 and 75–79 age groups for males and both genders, respectively. And it was higher in males than females in both years. Comparing 1990 and 2019 regarding YLDs rates showed higher rates in 1990 until the age of 69 years for males, and from 70 years and more, it was the other way around; however, for females, the YLDs rates were higher in 1990 until the age of 79 years and the pattern changed after the 80 years (Fig. 7).

**Figure 8:**
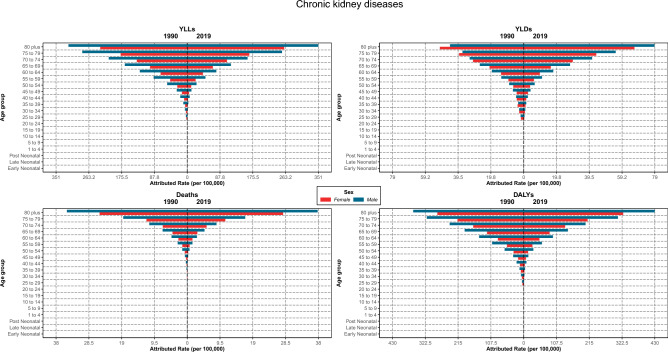
Burden rate due to chronic kidney diseases attributed to lead exposure by age groups and sex in Iran, 1990 vs 2019.

The burden of CKDs increased with aging for both genders, which was consistently higher in males than females. Moreover, the rates in each age group were higher in 1990 than in 2019 for both genders except for the 80-plus age group, demonstrating an inverted pattern. YLDs rate had an increasing trend with aging; its rates in males were always higher or equal to females except for 35–39 and 75 plus age groups. Furthermore, the rates of YLDs in the 65-plus age group in 2019 were higher than in 1990 in the same age group for both sexes, but other ages had an inverted pattern (Fig. 8).

**Figure 9:**
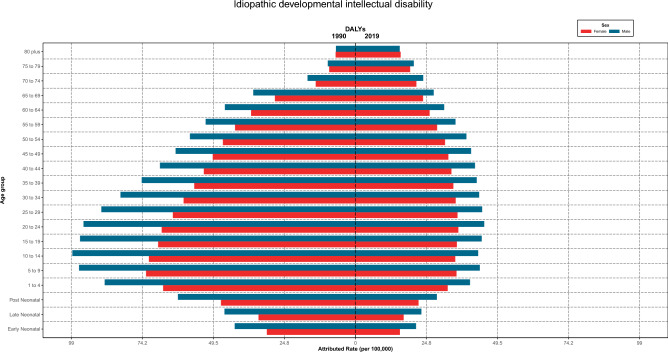
Burden rate due to idiopathic developmental intellectual disability attributed to lead exposure by age groups and sex in Iran, 1990 vs 2019.

IDID's DALYs rate increased until the age of 10–14 for males and 5–9 for females in 1990 and then decreased with aging; in 2019, it approximately increased until the age 20–24 and then decreased by aging. The rate was consistently higher in males than females except for the 80-plus age group in both years, which was roughly equal in both genders. Moreover, the rates demonstrated higher values in 1990 than in 2019 for both genders until the age of 69, while for the 70-plus age group, it was vice versa (Fig. 9).

## Discussion

The current study explored the burden of diseases attributed to LE in Iran at national and subnational scopes in 1990 and 2019. It was observed that the all-cause national and subnational ASDR, DALYs, YLL, and YLD rates attributed to LE had declining trends for both sexes during the past 30 years. These results were in line with a recent report on LE burden in North Africa and Middle East^22^. Furthermore, although divergences were present between the aforementioned epidemiologic indices in 1990 among different provinces, they converged more in 2019. Even though almost all of the inspected index rates declined across different SDI regions during the past 30 years, low and low-middle provinces had the highest all-cause ASDR, age-standardized DALYs, YLLs, and YLDs rates in both 1990 and 2019. In contrast, provinces in the top SDI quintile had the lowest rates in these years. Similar patterns were observed on a country-level scope in the Middle East and North Africa^22^.

The lead-related burden of CKD, IDID, and most sub-classes of CVD has decreased between 1990 and 2019 on a national scope. Although the same pattern can be observed in most provinces, a few epidemiological indices worsened in a handful of provinces, especially for CKD. For instance, ASDR was increased in Sistan and Baluchistan, Ilam, Ardebil, and East Azerbaijan. Most of these provinces either have lower SDI, such as Sistan and Baluchistan or are known as industrial provinces, such as East Azerbaijan. Low SDI has been reported to be related to higher blood lead levels^23^. In low SDI regions, the cultural and educational level of the population is lower than in high SDI regions^24^, causing potential unawareness and ignorance concerning the dangers of LE. Moreover, lead contained in old paints, pipes, and other objects might still contaminate the environment in low SDI regions as they have a limited budget to renovate and address these issues. Although industrial lead utilization has been regulated, lead is still used in many fields, such as automobile production, which can cause increased LE in industrial regions^25^.

Improved healthcare system and the advancement of medical practice are additional possible contributing factors to the observed decreased burden of lead-associated CVD and CKD. The treatment of IHD, stroke, and hypertensive heart diseases, as major subclasses of CVDs, depend upon timely intervention, adequately advanced medical equipment, and trained medical personnel^26^. A similar framework is also applicable to the treatment and care of CKD, as the disease requires meticulous acute and chronic care^27,28^. Improved education and information distribution have also made it possible to increase public awareness concerning the dangers of lead. However, the awareness levels remain low in rural and less developed regions, possibly contributing to the observed higher burden of lead-associated diseases in low SDI regions^29^.

Male individuals had higher lead-associated disease burdens than females. This observation is aligned with previous reports that male gender is associated with higher LE attributed disorders^22,30^. Gender related biological differences are reported to affect the dynamics and toxicity of metals in humans^31^. In addition, men make up the majority of the Iranian workforce, which could increase the probability of occupational contamination^22,32^. Regarding age groups, different age groups had similar lead-related disease patterns; all rates increased with aging, and the rates were lower in 2019 compared to 1990 in both genders. It is probable that more aged individuals potentially had more contact with lead in the past when the regulations were less strict. Moreover, the stored lead in soft tissue and bone can contaminate blood for long intervals, leading to increased exposure to the substance^33^. Finally, aging is a risk factor for many diseases, such as CVD and CKD^34–36^.

At the subnational level, a decrease in the burden of LE is evident from 1990 to 2019; in 1990, most provinces had high rates of ASDR, age-standardized DALYs, YLLs, and YLDs, but in 2019 the total burden diminished in most provinces and some rare provinces like south-east part of the country and some provinces like Hormozgan, Ardebil, Sistan and Baluchistan, South Khorasan experience the highest burden of LE that policymakers should give more attention. However, Tehran, the capital city of the country, suffers from air pollution due to its high population and industries^16^ but has the least burden attributed to LE overall and in CVDs, CKDs, mental disorders, and their subtypes. This might be due to several reasons but the high socioeconomic status and healthcare access in the capital of the country might have a role in this regards^37,38^.

In the current study, a reduction in the burden of diseases due to LE, estimated through deaths, DALYs, YLLs, and YLDs rates, was evident from 1990 to 2019, demonstrating overall decreasing trends over the mentioned time in Iran at national and subnational levels. This trend is also evident at the global level in many industrialized countries. The reason goes back to various actions like eliminating lead from household paints and phasing lead from gasoline, which helps reduce 80–90% of ambient lead in the air^39^. The same regulations and guidelines for worker safety to prevent occupational exposure have been implemented in Iran^16,40^. However, lead control regulations are passed in many countries and have reduced LE and its burden; enormous reservoirs nevertheless persist in old houses, paints, lead-acid batteries (increased demand with the use of automobiles, computers, and telecommunications), soil, dust, leaded gasoline used in aircraft, racing cars and etc.^41^. The abolishment of lead from industrial and non-industrial products is probably one of the most prominent factors in the observed reduction of SDI-independent lead induced burden.

Moreover, lead can be accumulated in the bone of exposed people and have different impacts on the body years after exposure. Also, in pregnant women can be a hazard for developing fetuses. Therefore LE will never go away, and the best approach is to reduce the exposure and prevent people from LE as much as possible^39,42^. In a study of the mean BLL in the Iranian population, they found a mean BLL of 6.41 μg/dL (5.96–6.87), which was higher than the CDC recommended level^43^, implying that the Iranian government has a long way to go before reaching the ideal amount of LE.

## Limitations

Despite using different practical approaches to assess and integrate the results of a variety of investigations, some limitations may indeed exist: first, Iran lacks a comprehensive registering program, which is crucial to the GBD study, to provide data regarding causes of deaths; and this could limit conclusions regarding ascribed mortality by causes. Second, the final results may be skewed due to differences in sample selection and burden measurement in prior surveys. The aforementioned limitations could be alleviated by future relevant national studies^44^. Moreover, all limitations to the GBD study would also apply to ours^12^.

## Conclusion

In conclusion, our study, which is the first national and subnational study to estimate the burden of disease attributes by environmental LE, found the possible various adverse health effects of LE on the body, including CVDs (the most common and severe), CKDs, and mental disorders. Thus, the investigation of LE national and subnational disease burden dynamics is highly valuable. In Iran, the exposure and burden attributed to LE have decreased from 1990 to 2019 regarding all causes of diseases and each disease subtype. Lead legislation in many countries, including Iran, has reduced the attributed burden; however, LE still occurs, and the burden in some provinces is significant. These data can give policymakers and health authorities a better understanding of whether previous restrictions were well implemented, allowing them to decide on new regulations based on source identification and exposure reduction or removal.

### Supplementary Information

Supplementary Legends.

Supplementary Figures.

Supplementary Information.

## Data Availability

The data are available with the corresponding author and can be reached on request.
