# Supplementary material for: Unveiling the lead exposure attributed burden in Iran from 1990 to 2019 through the lens of the Global Burden of Disease study 2019
Source: Sci Rep. 2024 Apr 15;14:8688. doi: 10.1038/s41598-024-58823-z (PMC11018826; doi:10.1038/s41598-024-58823-z)
Supplement: Supplementary file 1 — Supplementary Legends. [file 41598_2024_58823_MOESM1_ESM.docx]

**Legends of supplementary figures**

Supplementary figure 1. Ranking of provinces by age-standardized burden rate due to cardiovascular diseases attributed to lead exposure, 1990 vs 2019

Supplementary figure 2. Ranking of provinces by age-standardized burden rate due to chronic kidney diseases attributed to lead exposure, 1990 vs 2019

Supplementary figure 3. Ranking of provinces by age-standardized burden rate due to idiopathic developmental intellectual disability attributed to lead exposure, 1990 vs 2019

Supplementary figure 4. Distribution of provincial age-standardized burden rate due to all causes attributed to lead exposure by SDI quintiles, 1990 and 2019

*SDI: Socio-Demographic Index*

Supplementary figure 5. Provincial age-standardized attributed YLDs rate to lead exposure by SDI in 1990, 2000, 2010 and 2019

*YLDs: Years Lived with Disability; SDI: Socio-Demographic Index*

Supplementary figure 6. Provincial age-standardized attributed deaths rate to lead exposure by SDI in 1990, 2000, 2010 and 2019

*SDI: Socio-Demographic Index*

Supplementary figure 7. Provincial age-standardized attributed DALYs rate to lead exposure by SDI in 1990, 2000, 2010 and 2019

*DALYs: Disability-Adjusted Life Years; SDI: Socio-Demographic Index*

Supplementary figure 8. Distribution of provincial age-standardized burden rate due to peripheral artery disease attributed to lead exposure by SDI quintiles, 1990 and 2019

*SDI: Socio-Demographic Index*

Supplementary figure 9. Distribution of provincial age-standardized burden rate due to atrial fibrillation and flutter attributed to lead exposure by SDI quintiles, 1990 and 2019

*SDI: Socio-Demographic Index*

Supplementary figure 10. Distribution of provincial age-standardized burden rate due to rheumatic heart disease attributed to lead exposure by SDI quintiles, 1990 and 2019

*SDI: Socio-Demographic Index*

Supplementary figure 11. Distribution of provincial age-standardized burden rate due to ischemic stroke attributed to lead exposure by SDI quintiles, 1990 and 2019

*SDI: Socio-Demographic Index*

Supplementary figure 12. Distribution of provincial age-standardized burden rate due to subarachnoid hemorrhage attributed to lead exposure by SDI quintiles, 1990 and 2019

*SDI: Socio-Demographic Index*

Supplementary figure 13. Distribution of provincial age-standardized burden rate due to non−rheumatic valvular heart disease attributed to lead exposure by SDI quintiles, 1990 and 2019

*SDI: Socio-Demographic Index*

Supplementary figure 14. Distribution of provincial age-standardized burden rate due to cardiomyopathy and myocarditis attributed to lead exposure by SDI quintiles, 1990 and 2019

*SDI: Socio-Demographic Index*
