# Supplementary material for: Unveiling the lead exposure attributed burden in Iran from 1990 to 2019 through the lens of the Global Burden of Disease study 2019
Source: Sci Rep. 2024 Apr 15;14:8688. doi: 10.1038/s41598-024-58823-z (PMC11018826; doi:10.1038/s41598-024-58823-z)
Supplement: Supplementary file 2 — Supplementary Figures. [file 41598_2024_58823_MOESM2_ESM.pdf]

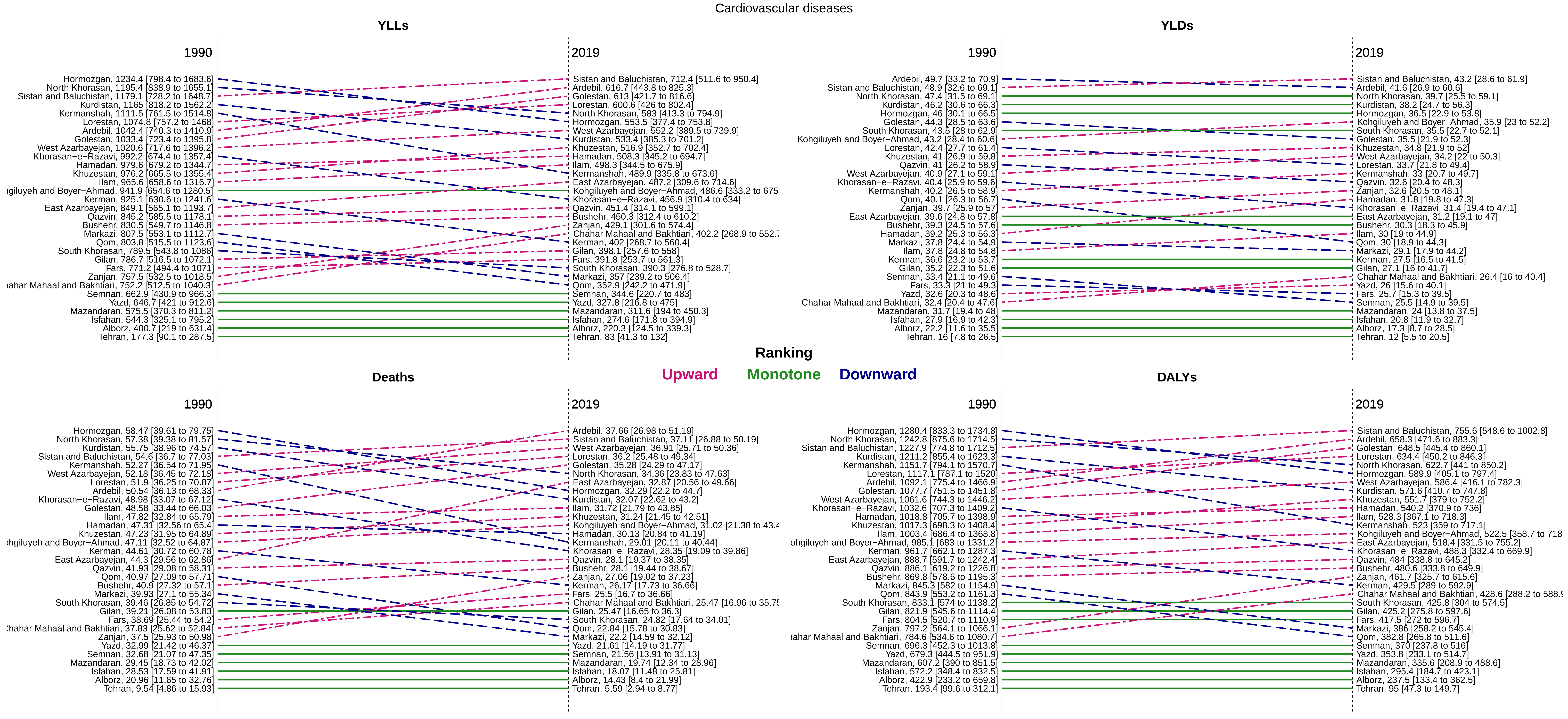

Cardiovascular diseases
YLLs
1990
2019
Hormozgan, 1234.4 [798.4 to 1683.6]
North Khorasan, 1195.4 [838.9 to 1655.1]
Sistan and Baluchistan, 1179.1 [728.2 to 1648.7]
Kurdistan, 1165 [818.2 to 1562.2]
Kermanshah, 1111.5 [761.5 to 1514.8]
Lorestan, 1074.8 [757.2 to 1468]
Ardebil, 1042.4 [740.3 to 1410.9]
Golestan, 1033.4 [723.4 to 1395.8]
West Azarbayejan, 1020.6 [717.6 to 1396.2]
Khorasan−e−Razavi, 992.2 [674.4 to 1357.4]
Hamadan, 979.6 [679.2 to 1344.7]
Khuzestan, 976.2 [665.5 to 1355.4]
Ilam, 965.6 [658.6 to 1316.7]
Kermann, 925.1 [630.6 to 1241.6]
East Azarbayejan, 849.1 [565.1 to 1193.7]
Qazvin, 845.2 [585.5 to 1178.1]
Bushehr, 830.5 [549.7 to 1146.8]
Markazi, 807.5 [553.1 to 1112.7]
Qom, 803.8 [515.5 to 1123.6]
South Khorasan, 789.5 [543.8 to 1086]
Gilan, 786.7 [516.5 to 1072.1]
Fars, 771.2 [494.4 to 1071]
Zanjan, 757.5 [532.5 to 1018.5]
Semnan, 662.9 [430.9 to 966.3]
Yazd, 646.7 [421 to 912.6]
Mazandaran, 575.5 [370.3 to 811.2]
Isfahan, 544.3 [325.1 to 795.2]
Alborz, 400.7 [219 to 631.4]
Tehran, 177.3 [90.1 to 287.5]
Sistan and Baluchistan, 712.4 [511.6 to 950.4]
Ardebil, 616.7 [443.8 to 825.3]
Golestan, 613 [421.7 to 816.6]
Lorestan, 600.6 [426 to 802.4]
North Khorasan, 583 [413.3 to 794.9]
Hormozgan, 553.5 [377.4 to 753.8]
West Azarbayejan, 552.2 [389.5 to 739.9]
Kurdistan, 533.4 [385.3 to 701.2]
Khuzestan, 516.9 [352.7 to 702.4]
Hamadan, 508.3 [345.2 to 694.7]
Ilam, 498.3 [344.5 to 675.9]
Kermanshah, 489.9 [335.8 to 673.6]
East Azarbayejan, 487.2 [309.6 to 714.6]
Khorasan−e−Razavi, 456.9 [310.4 to 634]
Qazvin, 451.4 [314.1 to 599.1]
Bushehr, 450.3 [312.4 to 610.2]
Zanjan, 429.1 [301.6 to 574.4]
Kerman, 402 [268.7 to 560.4]
Gilan, 398.1 [257.6 to 558]
Fars, 391.8 [253.7 to 561.3]
South Khorasan, 390.3 [276.8 to 528.7]
Markazi, 357 [239.2 to 506.4]
Qom, 352.9 [242.2 to 471.9]
Semnan, 344.6 [220.7 to 483]
Yazd, 327.8 [216.8 to 475]
Mazandaran, 311.6 [194 to 450.3]
Isfahan, 274.6 [171.8 to 394.9]
Alborz, 220.3 [124.5 to 339.3]
Tehran, 83 [41.3 to 132]
YLDs
1990
2019
Ardebil, 49.7 [33.2 to 70.9]
Sistan and Baluchistan, 48.9 [32.6 to 69.1]
North Khorasan, 47.4 [31.5 to 69.1]
Kurdistan, 46.2 [30.6 to 66.3]
Hormozgan, 46 [30.1 to 66.5]
Golestan, 44.3 [28.5 to 63.6]
South Khorasan, 43.5 [28 to 62.9]
Kohgiluyeh and Boyer−Ahmad, 43.2 [28.4 to 60.6]
Lorestan, 42.4 [27.7 to 61.4]
Khuzestan, 41 [26.9 to 59.8]
Qazvin, 41 [26.2 to 58.9]
West Azarbayejan, 40.9 [27.1 to 59.1]
Khorasan−e−Razavi, 40.4 [25.9 to 59.6]
Kermanshah, 40.2 [26.5 to 58.9]
Qom, 40.1 [26.3 to 56.7]
Zanjan, 39.7 [25.9 to 57]
East Azarbayejan, 39.6 [24.8 to 57.8]
Bushehr, 39.3 [24.5 to 57.6]
Hamadan, 39.2 [25.3 to 56.3]
Markazi, 37.8 [24.4 to 54.9]
Ilam, 37.8 [24.8 to 54.8]
Kerman, 36.6 [23.2 to 53.7]
Gilan, 35.2 [22.3 to 51.6]
Semnan, 33.4 [21.1 to 49.6]
Fars, 33.3 [21 to 49.3]
Yazd, 32.6 [20.3 to 48.6]
Chahar Mahaal and Bakhtiari, 32.4 [20.4 to 47.6]
Mazandaran, 31.7 [19.4 to 48]
Isfahan, 27.9 [16.9 to 42.3]
Alborz, 22.2 [11.6 to 35.5]
Tehran, 16 [7.8 to 26.5]
Sistan and Baluchistan, 43.2 [28.6 to 61.9]
Ardebil, 41.6 [26.9 to 60.6]
North Khorasan, 39.7 [25.5 to 59.1]
Kurdistan, 38.2 [24.7 to 56.3]
Hormozgan, 36.5 [22.9 to 53.8]
Kohgiluyeh and Boyer−Ahmad, 35.9 [23 to 52.2]
South Khorasan, 35.5 [22.7 to 52.1]
Golestan, 35.5 [21.9 to 52.3]
Khuzestan, 34.8 [21.9 to 52]
West Azarbayejan, 34.2 [22 to 50.3]
Lorestan, 33.7 [21.8 to 49.4]
Kermanshah, 33 [20.7 to 49.7]
Qazvin, 32.6 [20.4 to 48.3]
Zanjan, 32.6 [20.5 to 48.1]
Hamadan, 31.8 [19.8 to 47.3]
Khorasan−e−Razavi, 31.4 [19.4 to 47.1]
East Azarbayejan, 31.2 [19.1 to 47]
Bushehr, 30.3 [18.3 to 45.9]
Ilam, 30 [19 to 44.9]
Qom, 30 [18.9 to 44.3]
Markazi, 29.1 [17.9 to 44.2]
Kerman, 27.5 [16.5 to 41.5]
Gilan, 27.1 [16 to 41.7]
Chahar Mahaal and Bakhtiari, 26.4 [16 to 40.4]
Yazd, 26 [15.6 to 40.1]
Fars, 25.7 [15.3 to 39.5]
Semnan, 25.5 [14.9 to 39.5]
Mazandaran, 24 [13.8 to 37.5]
Isfahan, 20.8 [11.9 to 32.7]
Alborz, 17.3 [8.7 to 28.5]
Tehran, 12 [5.5 to 20.5]
Ranking
Upward
Monotone
Downward
Deaths
1990
2019
Hormozgan, 58.47 [39.61 to 79.75]
North Khorasan, 57.38 [39.38 to 81.57]
Kurdistan, 55.75 [38.96 to 74.57]
Sistan and Baluchistan, 54.6 [36.7 to 77.03]
Kermanshah, 52.27 [36.54 to 71.95]
West Azarbayejan, 52.18 [36.45 to 72.18]
Lorestan, 51.9 [36.25 to 70.87]
Ardebil, 50.54 [36.13 to 68.33]
Khorasan−e−Razavi, 48.98 [33.07 to 67.12]
Golestan, 48.58 [33.44 to 66.03]
Ilam, 47.82 [32.84 to 65.79]
Hamadan, 47.31 [32.56 to 65.4]
Khuzestan, 47.23 [31.95 to 64.89]
Kerman, 44.61 [30.72 to 60.78]
East Azarbayejan, 44.3 [29.56 to 62.86]
Qazvin, 41.93 [29.08 to 58.31]
Qom, 40.97 [27.09 to 57.71]
Bushehr, 40.9 [27.32 to 57.1]
Markazi, 39.93 [27.1 to 55.34]
South Khorasan, 39.46 [26.85 to 54.72]
Gilan, 39.21 [26.08 to 53.83]
Fars, 38.69 [25.44 to 54.2]
Zanjan, 37.5 [25.93 to 50.98]
Yazd, 32.99 [21.42 to 46.37]
Semnan, 32.68 [21.07 to 47.35]
Mazandaran, 29.45 [18.73 to 42.02]
Isfahan, 28.53 [17.59 to 41.91]
Alborz, 20.96 [11.65 to 32.76]
Tehran, 9.54 [4.86 to 15.93]
Ardebil, 37.66 [26.98 to 51.19]
Sistan and Baluchistan, 37.11 [26.88 to 50.19]
West Azarbayejan, 36.91 [25.71 to 50.36]
Lorestan, 36.2 [25.48 to 49.34]
Golestan, 35.28 [24.29 to 47.17]
North Khorasan, 34.36 [23.83 to 47.63]
East Azarbayejan, 32.87 [20.56 to 49.66]
Hormozgan, 32.29 [22.2 to 44.7]
Kurdistan, 32.07 [22.62 to 43.2]
Ilam, 31.72 [21.79 to 43.85]
Khuzestan, 31.24 [21.45 to 42.51]
Hamadan, 30.13 [20.84 to 41.19]
Kermanshah, 29.01 [20.11 to 40.44]
Khorasan−e−Razavi, 28.35 [19.09 to 39.86]
Qazvin, 28.1 [19.37 to 38.35]
Bushehr, 28.1 [19.44 to 38.67]
Zanjan, 27.06 [19.02 to 37.23]
Kerman, 26.17 [17.73 to 36.66]
Fars, 25.5 [16.7 to 36.66]
Gilan, 25.47 [16.65 to 36.3]
South Khorasan, 24.82 [17.64 to 34.01]
Qom, 22.84 [15.78 to 30.83]
Markazi, 22.2 [14.59 to 32.12]
Yazd, 21.61 [14.19 to 31.77]
Semnan, 21.56 [13.91 to 31.13]
Mazandaran, 19.74 [12.34 to 28.96]
Isfahan, 18.07 [11.48 to 25.81]
Alborz, 14.43 [8.4 to 21.99]
Tehran, 5.59 [2.94 to 8.77]
DALYs
1990
2019
Hormozgan, 1280.4 [833.3 to 1734.8]
North Khorasan, 1242.8 [875.6 to 1714.5]
Sistan and Baluchistan, 1227.9 [774.8 to 1712.5]
Kurdistan, 1211.2 [855.4 to 1623.3]
Kermanshah, 1151.7 [794.1 to 1570.7]
Lorestan, 1117.1 [787.1 to 1520]
Ardebil, 1092.1 [775.4 to 1466.9]
Golestan, 1077.7 [751.5 to 1451.8]
West Azarbayejan, 1061.6 [744.3 to 1446.2]
Khorasan−e−Razavi, 1032.6 [707.3 to 1409.2]
Hamadan, 1018.8 [705.7 to 1398.9]
Khuzestan, 1017.3 [698.3 to 1408.4]
Ilam, 1003.4 [686.4 to 1368.8]
Kerman, 961.7 [662.1 to 1287.3]
East Azarbayejan, 888.7 [591.7 to 1242.4]
Qazvin, 886.1 [619.2 to 1226.8]
Bushehr, 869.8 [578.6 to 1195.3]
Markazi, 845.3 [582 to 1154.9]
Qom, 843.9 [553.2 to 1161.3]
South Khorasan, 833.1 [574 to 1138.2]
Gilan, 821.9 [545.6 to 1114.4]
Fars, 804.5 [520.7 to 1110.9]
Zanjan, 797.2 [564.1 to 1066.1]
Semnan, 696.3 [452.3 to 1013.8]
Yazd, 679.3 [444.5 to 951.9]
Mazandaran, 607.2 [390 to 851.5]
Isfahan, 572.2 [348.4 to 832.5]
Alborz, 422.9 [233.2 to 659.8]
Tehran, 193.4 [99.6 to 312.1]
Sistan and Baluchistan, 755.6 [548.6 to 1002.8]
Ardebil, 658.3 [471.6 to 883.3]
Golestan, 648.5 [445.4 to 860.1]
Lorestan, 634.4 [450.2 to 846.3]
North Khorasan, 622.7 [441 to 850.2]
Hormozgan, 589.9 [405.1 to 797.4]
West Azarbayejan, 586.4 [416.1 to 782.3]
Kurdistan, 571.6 [410.7 to 747.8]
Khuzestan, 551.7 [379 to 752.2]
Hamadan, 540.2 [370.9 to 736]
Ilam, 528.3 [367.1 to 718.3]
Kermanshah, 523 [359 to 717.1]
East Azarbayejan, 518.4 [331.5 to 755.2]
Khorasan−e−Razavi, 488.3 [332.4 to 669.9]
Qazvin, 484 [338.8 to 645.2]
Bushehr, 480.6 [333.8 to 649.9]
Zanjan, 461.7 [325.7 to 615.6]
Kerman, 429.5 [289 to 592.9]
South Khorasan, 425.8 [304 to 574.5]
Gilan, 425.2 [275.8 to 597.6]
Fars, 417.5 [272 to 596.7]
Markazi, 386 [258.2 to 545.4]
Qom, 382.8 [265.8 to 511.6]
Semnan, 370 [237.8 to 516]
Yazd, 353.8 [233.1 to 514.7]
Mazandaran, 335.6 [208.9 to 488.6]
Isfahan, 295.4 [184.7 to 423.1]
Alborz, 237.5 [133.4 to 362.5]
Tehran, 95 [47.3 to 149.7]

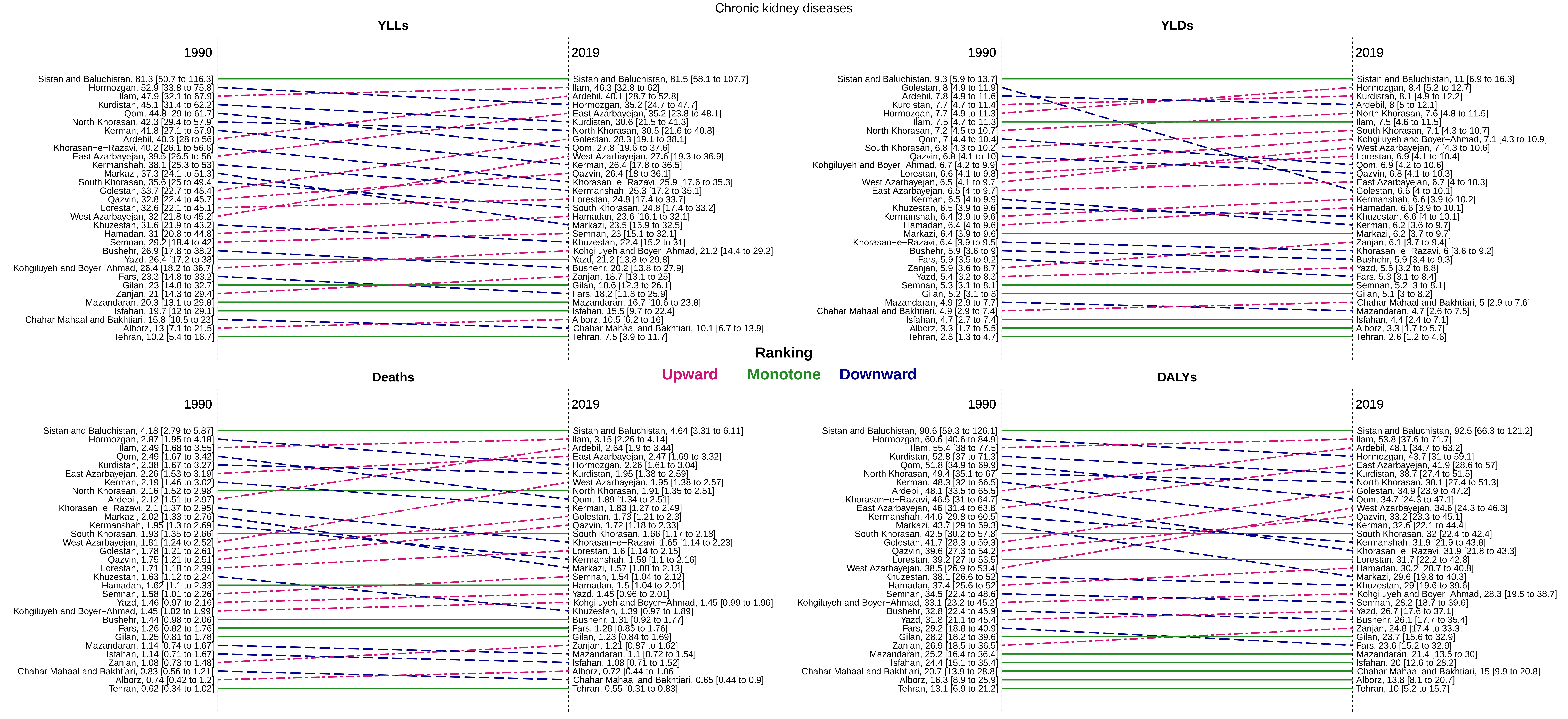
Chronic kidney diseases
YLLs
1990
2019
Sistan and Baluchistan, 81.3 [50.7 to 116.3]
Hormozgan, 52.9 [33.8 to 75.8]
Ilam, 47.9 [32.1 to 67.9]
Kurdistan, 45.1 [31.4 to 62.2]
Qom, 44.8 [29 to 61.7]
North Khorasan, 42.3 [29.4 to 57.9]
Kerman, 41.8 [27.1 to 57.9]
Ardebil, 40.3 [28 to 56]
Khorasan−e−Razavi, 40.2 [26.1 to 56.6]
East Azarbayejan, 39.5 [26.5 to 56]
Kermanshah, 38.1 [25.3 to 53]
Markazi, 37.3 [24.1 to 51.3]
South Khorasan, 35.6 [25 to 49.4]
Golestan, 33.7 [22.7 to 48.4]
Qazvin, 32.8 [22.4 to 45.7]
Lorestan, 32.6 [22.1 to 45.1]
West Azarbayejan, 32 [21.8 to 45.2]
Khuzestan, 31.6 [21.9 to 43.2]
Hamadan, 31 [20.8 to 44.8]
Semnan, 29.2 [18.4 to 42]
Bushehr, 26.9 [17.8 to 38.2]
Yazd, 26.4 [17.2 to 38]
Kohgiluyeh and Boyer−Ahmad, 26.4 [18.2 to 36.7]
Fars, 23.3 [14.8 to 33.2]
Gilan, 23 [14.8 to 32.7]
Zanjan, 21 [14.3 to 29.4]
Mazandaran, 20.3 [13.1 to 29.8]
Isfahan, 19.7 [12 to 29.1]
Chahar Mahaal and Bakhtiari, 15.8 [10.5 to 23]
Alborz, 13 [7.1 to 21.5]
Tehran, 10.2 [5.4 to 16.7]
Sistan and Baluchistan, 81.5 [58.1 to 107.7]
Ilam, 46.3 [32.8 to 62]
Ardebil, 40.1 [28.7 to 52.8]
Hormozgan, 35.2 [24.7 to 47.7]
East Azarbayejan, 35.2 [23.8 to 48.1]
Kurdistan, 30.6 [21.5 to 41.3]
North Khorasan, 30.5 [21.6 to 40.8]
Golestan, 28.3 [19.1 to 38.1]
Qom, 27.8 [19.6 to 37.6]
West Azarbayejan, 27.6 [19.3 to 36.9]
Kerman, 26.4 [17.8 to 36.5]
Qazvin, 26.4 [18 to 36.1]
Khorasan−e−Razavi, 25.9 [17.6 to 35.3]
Kermanshah, 25.3 [17.2 to 35.1]
Lorestan, 24.8 [17.4 to 33.7]
South Khorasan, 24.8 [17.4 to 33.2]
Hamadan, 23.6 [16.1 to 32.1]
Markazi, 23.5 [15.9 to 32.5]
Semnan, 23 [15.1 to 32.1]
Khuzestan, 22.4 [15.2 to 31]
Kohgiluyeh and Boyer−Ahmad, 21.2 [14.4 to 29.2]
Yazd, 21.2 [13.8 to 29.8]
Bushehr, 20.2 [13.8 to 27.9]
Zanjan, 18.7 [13.1 to 25]
Gilan, 18.6 [12.3 to 26.1]
Fars, 18.2 [11.8 to 25.9]
Mazandaran, 16.7 [10.6 to 23.8]
Isfahan, 15.5 [9.7 to 22.4]
Alborz, 10.5 [6.2 to 16]
Chahar Mahaal and Bakhtiari, 10.1 [6.7 to 13.9]
Tehran, 7.5 [3.9 to 11.7]
YLDs
1990
2019
Sistan and Baluchistan, 9.3 [5.9 to 13.7]
Golestan, 8 [4.9 to 11.9]
Ardebil, 7.8 [4.9 to 11.6]
Kurdistan, 7.7 [4.7 to 11.4]
Hormozgan, 7.7 [4.9 to 11.3]
Ilam, 7.5 [4.7 to 11.3]
North Khorasan, 7.2 [4.5 to 10.7]
Qom, 7 [4.4 to 10.4]
South Khorasan, 6.8 [4.3 to 10.2]
Qazvin, 6.8 [4.1 to 10]
Kohgiluyeh and Boyer−Ahmad, 6.7 [4.2 to 9.9]
Lorestan, 6.6 [4.1 to 9.8]
West Azarbayejan, 6.5 [4.1 to 9.7]
East Azarbayejan, 6.5 [4 to 9.7]
Kerman, 6.5 [4 to 9.9]
Khuzestan, 6.5 [3.9 to 9.6]
Kermanshah, 6.4 [3.9 to 9.6]
Hamadan, 6.4 [4 to 9.6]
Markazi, 6.4 [3.9 to 9.6]
Khorasan−e−Razavi, 6.4 [3.9 to 9.5]
Bushehr, 5.9 [3.6 to 9]
Fars, 5.9 [3.5 to 9.2]
Zanjan, 5.9 [3.6 to 8.7]
Yazd, 5.4 [3.2 to 8.3]
Semnan, 5.3 [3.1 to 8.1]
Gilan, 5.1 [3.1 to 8]
Mazandaran, 4.9 [2.9 to 7.7]
Chahar Mahaal and Bakhtiari, 4.9 [2.9 to 7.4]
Isfahan, 4.7 [2.7 to 7.4]
Alborz, 3.3 [1.7 to 5.5]
Tehran, 2.8 [1.3 to 4.7]
Sistan and Baluchistan, 11 [6.9 to 16.3]
Hormozgan, 8.4 [5.2 to 12.7]
Kurdistan, 8.1 [4.9 to 12.2]
Ardebil, 8 [5 to 12.1]
North Khorasan, 7.6 [4.8 to 11.5]
Ilam, 7.5 [4.6 to 11.5]
South Khorasan, 7.1 [4.3 to 10.7]
Kohgiluyeh and Boyer−Ahmad, 7.1 [4.3 to 10.9]
West Azarbayejan, 7 [4.3 to 10.6]
Lorestan, 6.9 [4.1 to 10.4]
Qom, 6.9 [4.2 to 10.6]
Qazvin, 6.8 [4.1 to 10.3]
East Azarbayejan, 6.7 [4 to 10.3]
Golestan, 6.6 [4 to 10.1]
Kermanshah, 6.6 [3.9 to 10.2]
Hamadan, 6.6 [3.9 to 10.1]
Khuzestan, 6.6 [4 to 10.1]
Kerman, 6.2 [3.6 to 9.7]
Markazi, 6.2 [3.7 to 9.7]
Zanjan, 6.1 [3.7 to 9.4]
Khorasan−e−Razavi, 6 [3.6 to 9.2]
Bushehr, 5.9 [3.4 to 9.3]
Yazd, 5.5 [3.2 to 8.8]
Fars, 5.3 [3.1 to 8.4]
Semnan, 5.2 [3 to 8.1]
Gilan, 5.1 [3 to 8.2]
Chahar Mahaal and Bakhtiari, 5 [2.9 to 7.6]
Mazandaran, 4.7 [2.6 to 7.5]
Isfahan, 4.4 [2.4 to 7.1]
Alborz, 3.3 [1.7 to 5.7]
Tehran, 2.6 [1.2 to 4.6]
Ranking
Upward
Monotone
Downward
Deaths
1990
2019
Sistan and Baluchistan, 4.18 [2.79 to 5.87]
Hormozgan, 2.87 [1.95 to 4.18]
Ilam, 2.49 [1.68 to 3.55]
Qom, 2.49 [1.67 to 3.42]
Kurdistan, 2.38 [1.67 to 3.27]
East Azarbayejan, 2.26 [1.53 to 3.19]
Kerman, 2.19 [1.46 to 3.02]
North Khorasan, 2.16 [1.52 to 2.98]
Ardebil, 2.12 [1.51 to 2.97]
Khorasan−e−Razavi, 2.1 [1.37 to 2.95]
Markazi, 2.02 [1.33 to 2.76]
Kermanshah, 1.95 [1.3 to 2.69]
South Khorasan, 1.93 [1.35 to 2.66]
West Azarbayejan, 1.81 [1.24 to 2.52]
Golestan, 1.78 [1.21 to 2.61]
Qazvin, 1.75 [1.21 to 2.51]
Lorestan, 1.71 [1.18 to 2.39]
Khuzestan, 1.63 [1.12 to 2.24]
Hamadan, 1.62 [1.1 to 2.33]
Semnan, 1.58 [1.01 to 2.26]
Yazd, 1.46 [0.97 to 2.16]
Kohgiluyeh and Boyer−Ahmad, 1.45 [1.02 to 1.99]
Bushehr, 1.44 [0.98 to 2.06]
Fars, 1.26 [0.82 to 1.76]
Gilan, 1.25 [0.81 to 1.78]
Mazandaran, 1.14 [0.74 to 1.67]
Isfahan, 1.14 [0.71 to 1.67]
Zanjan, 1.08 [0.73 to 1.48]
Chahar Mahaal and Bakhtiari, 0.83 [0.56 to 1.21]
Alborz, 0.74 [0.42 to 1.2]
Tehran, 0.62 [0.34 to 1.02]
Sistan and Baluchistan, 4.64 [3.31 to 6.11]
Ilam, 3.15 [2.26 to 4.14]
Ardebil, 2.64 [1.9 to 3.44]
East Azarbayejan, 2.47 [1.69 to 3.32]
Hormozgan, 2.26 [1.61 to 3.04]
Kurdistan, 1.95 [1.38 to 2.59]
West Azarbayejan, 1.95 [1.38 to 2.57]
North Khorasan, 1.91 [1.35 to 2.51]
Qom, 1.89 [1.34 to 2.51]
Kerman, 1.83 [1.27 to 2.49]
Golestan, 1.73 [1.21 to 2.3]
Qazvin, 1.72 [1.18 to 2.33]
South Khorasan, 1.66 [1.17 to 2.18]
Khorasan−e−Razavi, 1.65 [1.14 to 2.23]
Lorestan, 1.6 [1.14 to 2.15]
Kermanshah, 1.59 [1.1 to 2.16]
Markazi, 1.57 [1.08 to 2.13]
Semnan, 1.54 [1.04 to 2.12]
Hamadan, 1.5 [1.04 to 2.01]
Yazd, 1.45 [0.96 to 2.01]
Kohgiluyeh and Boyer−Ahmad, 1.45 [0.99 to 1.96]
Khuzestan, 1.39 [0.97 to 1.89]
Bushehr, 1.31 [0.92 to 1.77]
Fars, 1.28 [0.85 to 1.76]
Gilan, 1.23 [0.84 to 1.69]
Zanjan, 1.21 [0.87 to 1.62]
Mazandaran, 1.1 [0.72 to 1.54]
Isfahan, 1.08 [0.71 to 1.52]
Alborz, 0.72 [0.44 to 1.06]
Chahar Mahaal and Bakhtiari, 0.65 [0.44 to 0.9]
Tehran, 0.55 [0.31 to 0.83]
DALYs
1990
2019
Sistan and Baluchistan, 90.6 [59.3 to 126.1]
Hormozgan, 60.6 [40.6 to 84.9]
Ilam, 55.4 [38 to 77.5]
Kurdistan, 52.8 [37 to 71.3]
Qom, 51.8 [34.9 to 69.9]
North Khorasan, 49.4 [35.1 to 67]
Kerman, 48.3 [32 to 66.5]
Ardebil, 48.1 [33.5 to 65.5]
Khorasan−e−Razavi, 46.5 [31 to 64.7]
East Azarbayejan, 46 [31.4 to 63.8]
Kermanshah, 44.6 [29.8 to 60.5]
Markazi, 43.7 [29 to 59.3]
South Khorasan, 42.5 [30.2 to 57.8]
Golestan, 41.7 [28.3 to 59.3]
Qazvin, 39.6 [27.3 to 54.2]
Lorestan, 39.2 [27 to 53.5]
West Azarbayejan, 38.5 [26.9 to 53.4]
Khuzestan, 38.1 [26.6 to 52]
Hamadan, 37.4 [25.6 to 52]
Semnan, 34.5 [22.4 to 48.6]
Kohgiluyeh and Boyer−Ahmad, 33.1 [23.2 to 45.2]
Bushehr, 32.8 [22.4 to 45.9]
Yazd, 31.8 [21.1 to 45.4]
Fars, 29.2 [18.8 to 40.9]
Gilan, 28.2 [18.2 to 39.6]
Zanjan, 26.9 [18.5 to 36.5]
Mazandaran, 25.2 [16.4 to 36.4]
Isfahan, 24.4 [15.1 to 35.4]
Chahar Mahaal and Bakhtiari, 20.7 [13.9 to 28.8]
Alborz, 16.3 [8.9 to 25.9]
Tehran, 13.1 [6.9 to 21.2]
Sistan and Baluchistan, 92.5 [66.3 to 121.2]
Ilam, 53.8 [37.6 to 71.7]
Ardebil, 48.1 [34.7 to 63.2]
Hormozgan, 43.7 [31 to 59.1]
East Azarbayejan, 41.9 [28.6 to 57]
Kurdistan, 38.7 [27.4 to 51.5]
North Khorasan, 38.1 [27.4 to 51.3]
Golestan, 34.9 [23.9 to 47.2]
Qom, 34.7 [24.3 to 47.1]
West Azarbayejan, 34.6 [24.3 to 46.3]
Qazvin, 33.2 [23.3 to 45.1]
Kerman, 32.6 [22.1 to 44.4]
South Khorasan, 32 [22.4 to 42.4]
Kermanshah, 31.9 [21.9 to 43.8]
Khorasan−e−Razavi, 31.9 [21.8 to 43.3]
Lorestan, 31.7 [22.2 to 42.8]
Hamadan, 30.2 [20.7 to 40.8]
Markazi, 29.6 [19.8 to 40.3]
Khuzestan, 29 [19.6 to 39.6]
Kohgiluyeh and Boyer−Ahmad, 28.3 [19.5 to 38.7]
Semnan, 28.2 [18.7 to 39.6]
Yazd, 26.7 [17.6 to 37.1]
Bushehr, 26.1 [17.7 to 35.4]
Zanjan, 24.8 [17.4 to 33.3]
Gilan, 23.7 [15.6 to 32.9]
Fars, 23.6 [15.2 to 32.9]
Mazandaran, 21.4 [13.5 to 30]
Isfahan, 20 [12.6 to 28.2]
Chahar Mahaal and Bakhtiari, 15 [9.9 to 20.8]
Alborz, 13.8 [8.1 to 20.7]
Tehran, 10 [5.2 to 15.7]

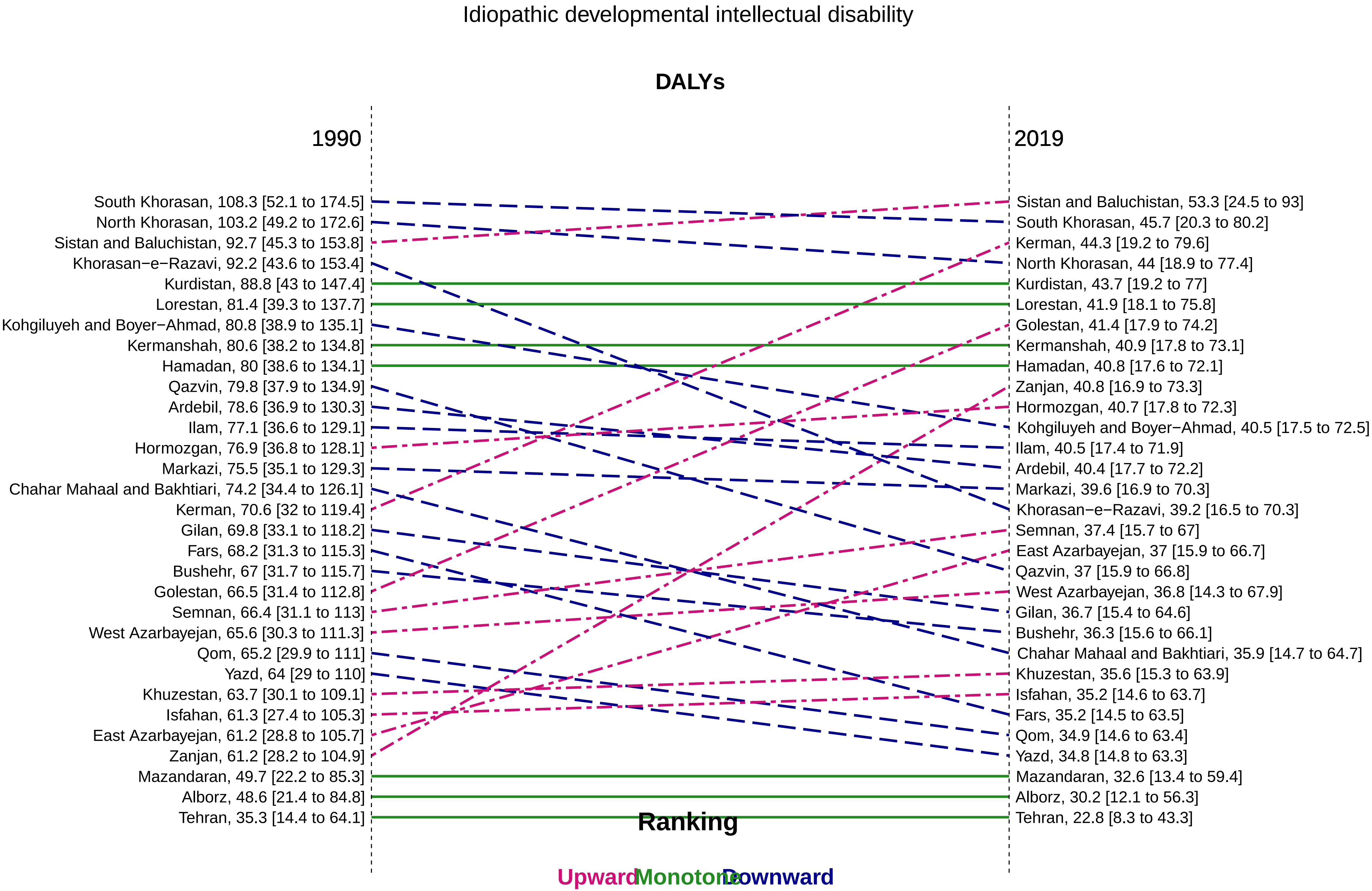

Idiopathic developmental intellectual disability
DALYs
1990
2019
South Khorasan, 108.3 [52.1 to 174.5]
North Khorasan, 103.2 [49.2 to 172.6]
Sistan and Baluchistan, 92.7 [45.3 to 153.8]
Khorasan−e−Razavi, 92.2 [43.6 to 153.4]
Kurdistan, 88.8 [43 to 147.4]
Lorestan, 81.4 [39.3 to 137.7]
Kohgiluyeh and Boyer−Ahmad, 80.8 [38.9 to 135.1]
Kermanshah, 80.6 [38.2 to 134.8]
Hamadan, 80 [38.6 to 134.1]
Qazvin, 79.8 [37.9 to 134.9]
Ardebil, 78.6 [36.9 to 130.3]
Ilam, 77.1 [36.6 to 129.1]
Hormozgan, 76.9 [36.8 to 128.1]
Markazi, 75.5 [35.1 to 129.3]
Chahar Mahaal and Bakhtiari, 74.2 [34.4 to 126.1]
Kerman, 70.6 [32 to 119.4]
Gilan, 69.8 [33.1 to 118.2]
Fars, 68.2 [31.3 to 115.3]
Bushehr, 67 [31.7 to 115.7]
Golestan, 66.5 [31.4 to 112.8]
Semnan, 66.4 [31.1 to 113]
West Azarbayejan, 65.6 [30.3 to 111.3]
Qom, 65.2 [29.9 to 111]
Yazd, 64 [29 to 110]
Khuzestan, 63.7 [30.1 to 109.1]
Isfahan, 61.3 [27.4 to 105.3]
East Azarbayejan, 61.2 [28.8 to 105.7]
Zanjan, 61.2 [28.2 to 104.9]
Mazandaran, 49.7 [22.2 to 85.3]
Alborz, 48.6 [21.4 to 84.8]
Tehran, 35.3 [14.4 to 64.1]
Sistan and Baluchistan, 53.3 [24.5 to 93]
South Khorasan, 45.7 [20.3 to 80.2]
Kerman, 44.3 [19.2 to 79.6]
North Khorasan, 44 [18.9 to 77.4]
Kurdistan, 43.7 [19.2 to 77]
Lorestan, 41.9 [18.1 to 75.8]
Golestan, 41.4 [17.9 to 74.2]
Kermanshah, 40.9 [17.8 to 73.1]
Hamadan, 40.8 [17.6 to 72.1]
Zanjan, 40.8 [16.9 to 73.3]
Hormozgan, 40.7 [17.8 to 72.3]
Kohgiluyeh and Boyer−Ahmad, 40.5 [17.5 to 72.5]
Ilam, 40.5 [17.4 to 71.9]
Ardebil, 40.4 [17.7 to 72.2]
Markazi, 39.6 [16.9 to 70.3]
Khorasan−e−Razavi, 39.2 [16.5 to 70.3]
Semnan, 37.4 [15.7 to 67]
East Azarbayejan, 37 [15.9 to 66.7]
Qazvin, 37 [15.9 to 66.8]
West Azarbayejan, 36.8 [14.3 to 67.9]
Gilan, 36.7 [15.4 to 64.6]
Bushehr, 36.3 [15.6 to 66.1]
Chahar Mahaal and Bakhtiari, 35.9 [14.7 to 64.7]
Khuzestan, 35.6 [15.3 to 63.9]
Isfahan, 35.2 [14.6 to 63.7]
Fars, 35.2 [14.5 to 63.5]
Qom, 34.9 [14.6 to 63.4]
Yazd, 34.8 [14.8 to 63.3]
Mazandaran, 32.6 [13.4 to 59.4]
Alborz, 30.2 [12.1 to 56.3]
Tehran, 22.8 [8.3 to 43.3]
Ranking
Upward
Monotone
Downward

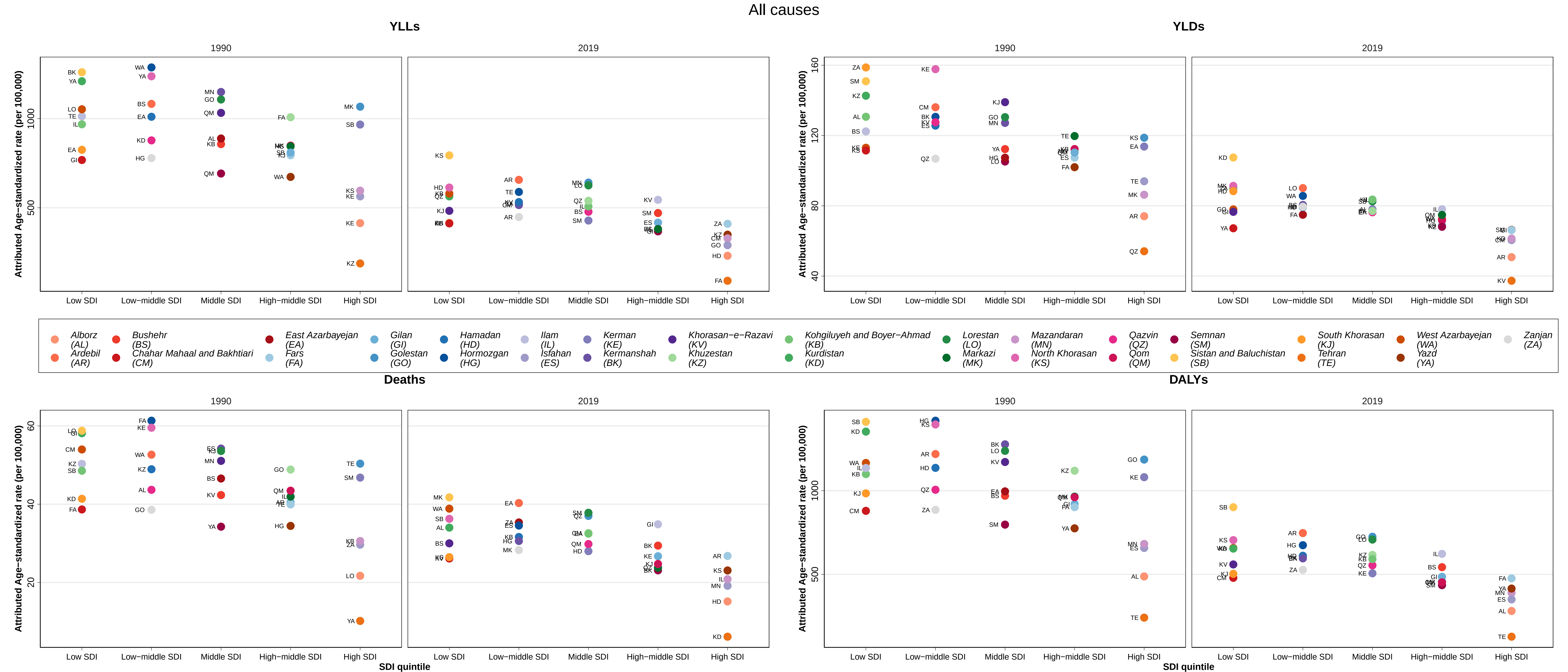

All causes
YLLs
YLDs
Deaths
DALYs
1990
2019
Attributed Age−standardized rate (per 100,000)
Low SDI
Low−middle SDI
Middle SDI
High−middle SDI
High SDI
SDI quintile
Alborz (AL)
Ardebil (AR)
Bushehr (BS)
Chahar Mahaal and Bakhtiari (CM)
East Azarbayejan (EA)
Fars (FA)
Gilan (GI)
Golestan (GO)
Hamadan (HD)
Hormozgan (HG)
Ilam (IL)
Isfahan (ES)
Kerman (KE)
Kermanshah (BK)
Khorasan−e−Razavi (KV)
Khuzestan (KZ)
Kohgiluyeh and Boyer−Ahmad (KB)
Kurdistan (KD)
Lorestan (LO)
Markazi (MK)
Mazandaran (MN)
North Khorasan (KS)
Qazvin (QZ)
Qom (QM)
Semnan (SM)
Sistan and Baluchistan (SB)
South Khorasan (KJ)
Tehran (TE)
West Azarbayejan (WA)
Yazd (YA)
Zanjan (ZA)

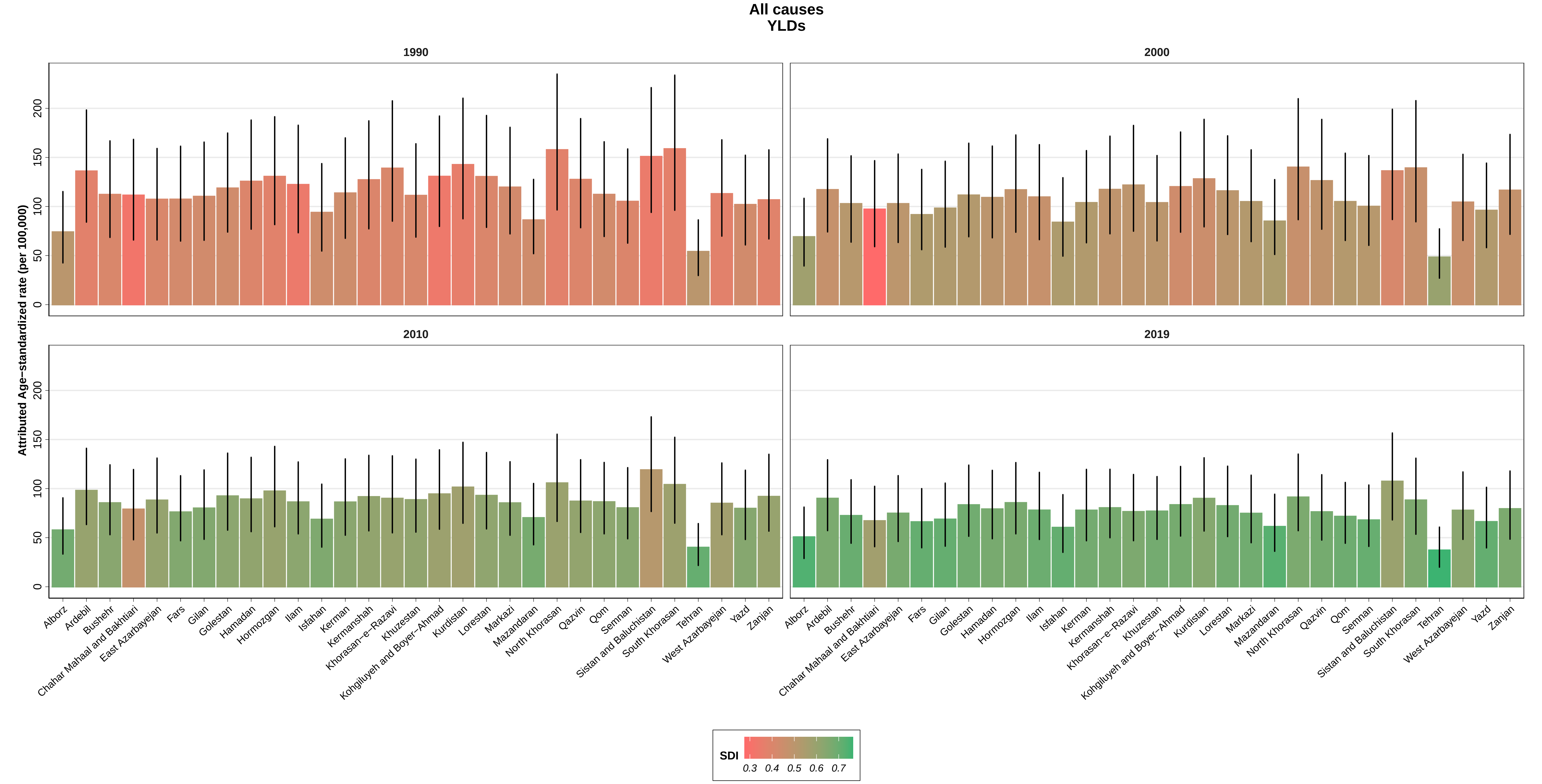
All causes
YLDs
1990
2000
2010
2019
Attributed Age−standardized rate (per 100,000)
0
50
100
150
200
Alborz
Ardebil
Bushehr
Chahar Mahaal and Bakhtiari
East Azarbayejan
Fars
Gilan
Golestan
Hamadan
Hormozgan
Ilam
Isfahan
Kerman
Kermanshah
Khorasan−e−Razavi
Khuzestan
Kohgiluyeh and Boyer−Ahmad
Kurdistan
Lorestan
Markazi
Mazandaran
North Khorasan
Qazvin
Qom
Semnan
Sistan and Baluchistan
South Khorasan
Tehran
West Azarbayejan
Yazd
Zanjan
SDI
0.3
0.4
0.5
0.6
0.7

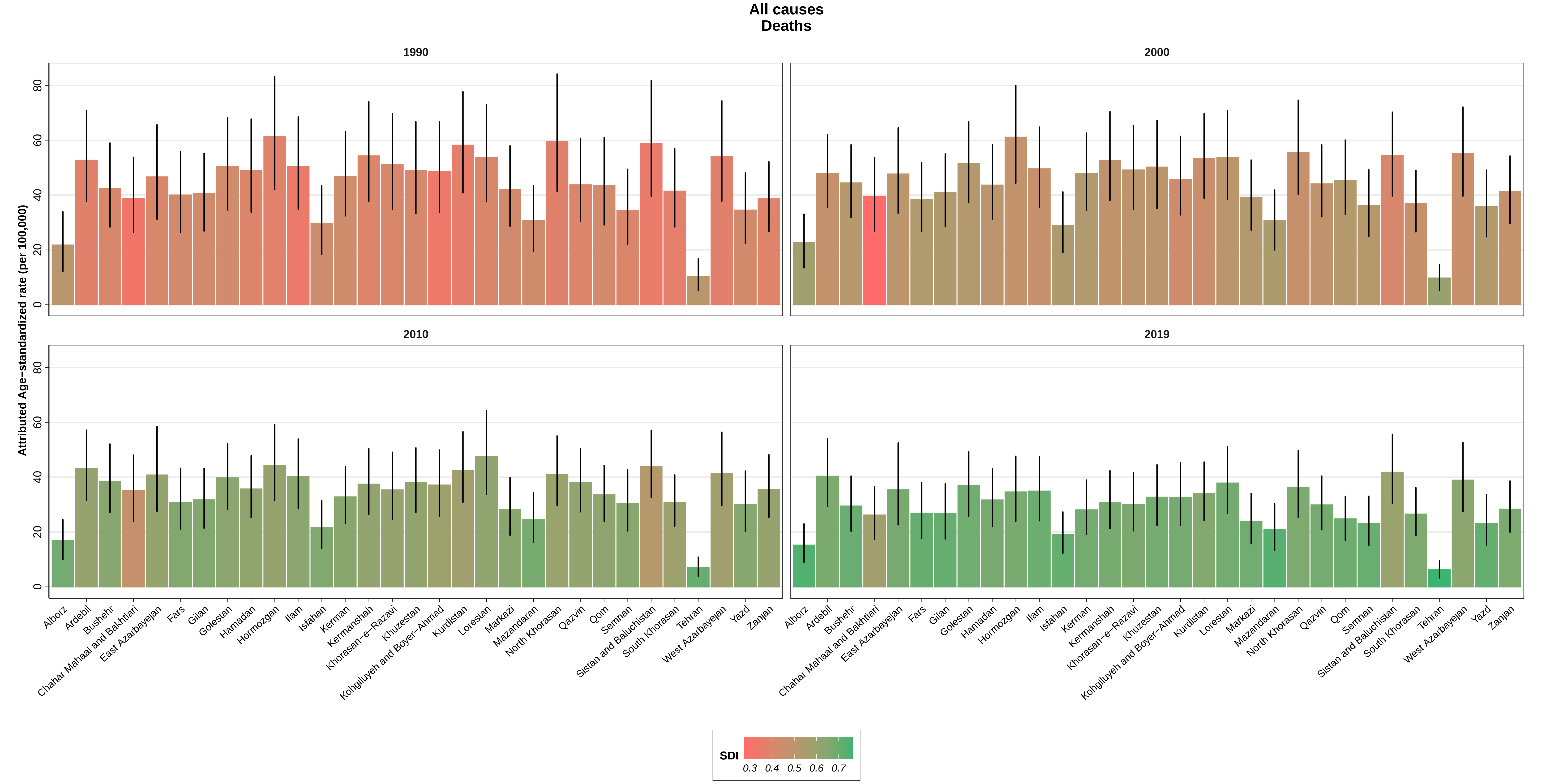
All causes
Deaths
1990
2000
2010
2019
Attributed Age−standardized rate (per 100,000)
0
20
40
60
80
Alborz
Ardebil
Bushehr
Chahar Mahaal and Bakhtiari
East Azarbayejan
Fars
Gilan
Golestan
Hamadan
Hormozgan
Ilam
Isfahan
Kerman
Kermanshah
Khorasan−e−Razavi
Khuzestan
Kohgiluyeh and Boyer−Ahmad
Kurdistan
Lorestan
Markazi
Mazandaran
North Khorasan
Qazvin
Qom
Semnan
Sistan and Baluchistan
South Khorasan
Tehran
West Azarbayejan
Yazd
Zanjan
SDI
0.3
0.4
0.5
0.6
0.7

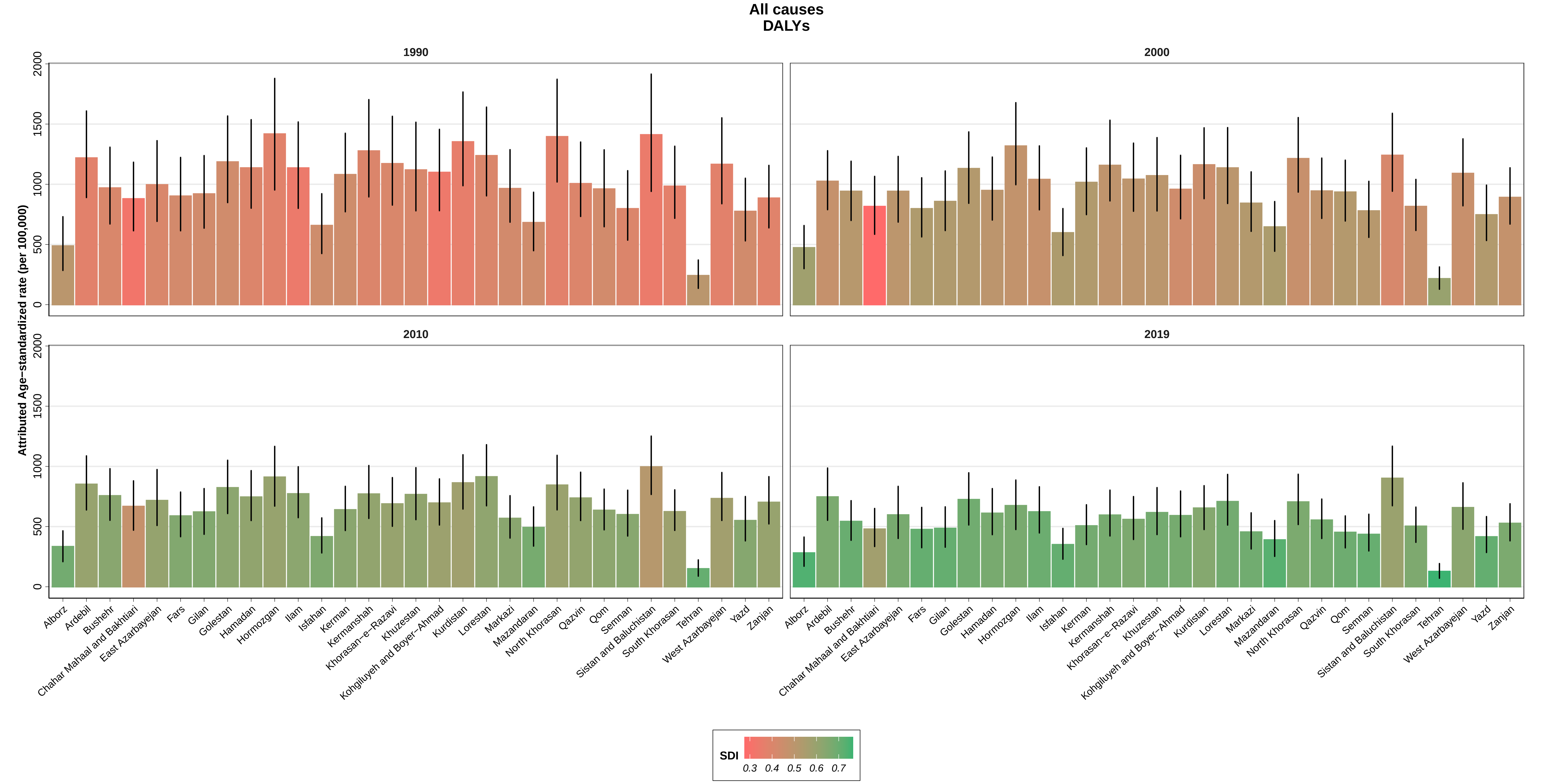
All causes
DALYs
1990
2000
2010
2019
Attributed Age−standardized rate (per 100,000)
0
500
1000
1500
2000
Alborz
Ardebil
Bushehr
Chahar Mahaal and Bakhtiari
East Azarbayejan
Fars
Gilan
Golestan
Hamadan
Hormozgan
Ilam
Isfahan
Kerman
Kermanshah
Khorasan−e−Razavi
Khuzestan
Kohgiluyeh and Boyer−Ahmad
Kurdistan
Lorestan
Markazi
Mazandaran
North Khorasan
Qazvin
Qom
Semnan
Sistan and Baluchistan
South Khorasan
Tehran
West Azarbayejan
Yazd
Zanjan
SDI
0.3
0.4
0.5
0.6
0.7

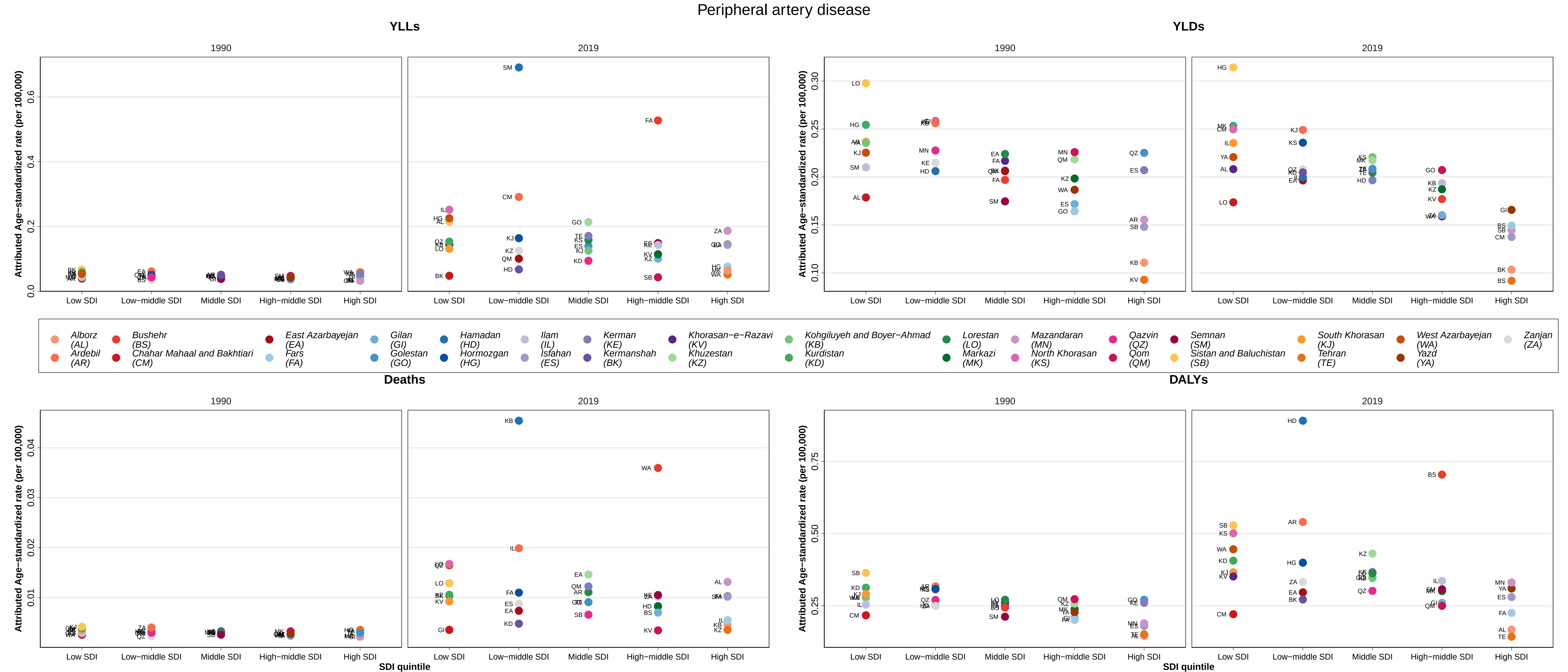
Peripheral artery disease
YLLs
YLDs
Deaths
DALYs
1990
2019
Attributed Age−standardized rate (per 100,000)
SDI quintile
Low SDI
Low−middle SDI
Middle SDI
High−middle SDI
High SDI
Alborz (AL)
Ardebil (AR)
Bushehr (BS)
Chahar Mahaal and Bakhtiari (CM)
East Azarbayejan (EA)
Fars (FA)
Gilan (GI)
Golestan (GO)
Hamadan (HD)
Hormozgan (HG)
Ilam (IL)
Isfahan (ES)
Kerman (KE)
Kermanshah (BK)
Khorasan−e−Razavi (KV)
Khuzestan (KZ)
Kohgiluyeh and Boyer−Ahmad (KB)
Kurdistan (KD)
Lorestan (LO)
Markazi (MK)
Mazandaran (MN)
North Khorasan (KS)
Qazvin (QZ)
Qom (QM)
Semnan (SM)
Sistan and Baluchistan (SB)
South Khorasan (KJ)
Tehran (TE)
West Azarbayejan (WA)
Yazd (YA)
Zanjan (ZA)

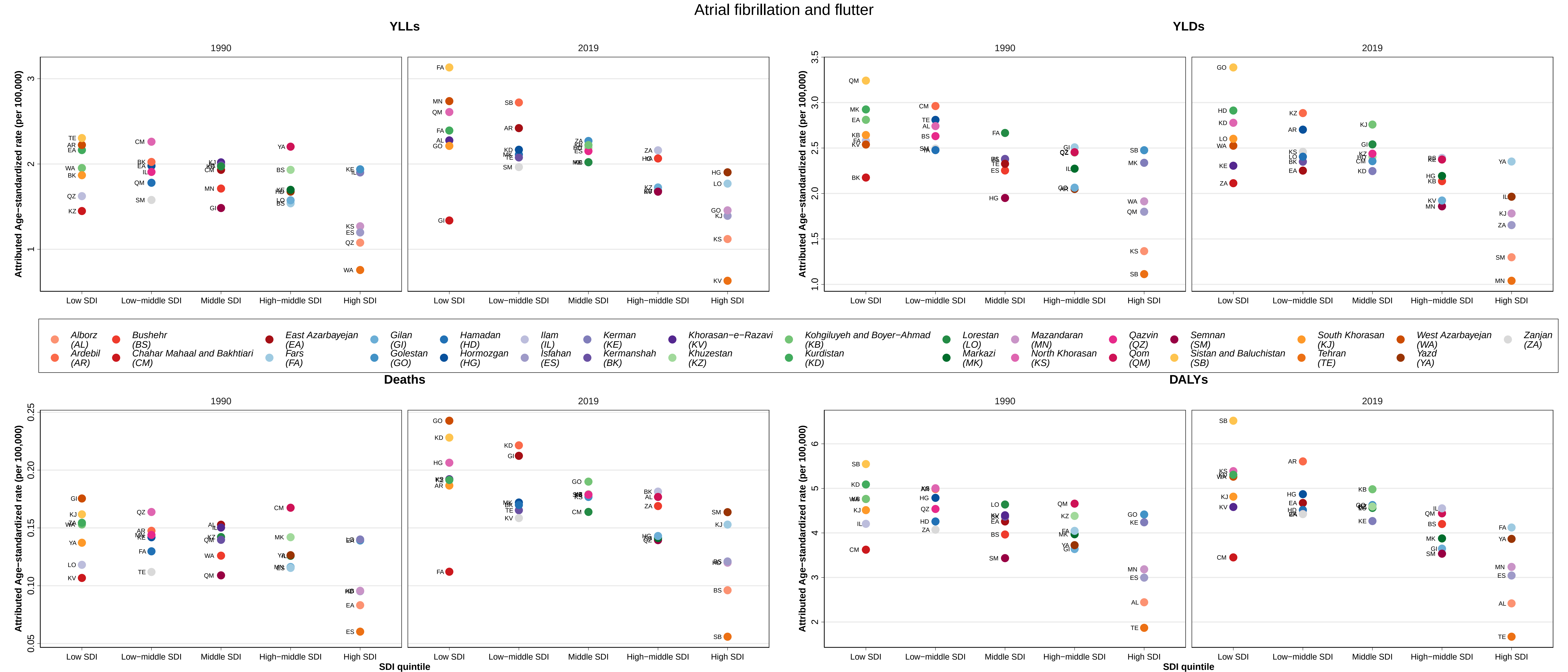

Atrial fibrillation and flutter
YLLs
YLDs
Deaths
DALYs
1990
2019
Attributed Age−standardized rate (per 100,000)
Low SDI
Low−middle SDI
Middle SDI
High−middle SDI
High SDI
SDI quintile
Alborz (AL)
Ardebil (AR)
Bushehr (BS)
Chahar Mahaal and Bakhtiari (CM)
East Azarbayejan (EA)
Fars (FA)
Gilan (GI)
Golestan (GO)
Hamadan (HD)
Hormozgan (HG)
Ilam (IL)
Isfahan (ES)
Kerman (KE)
Kermanshah (BK)
Khorasan−e−Razavi (KV)
Khuzestan (KZ)
Kohgiluyeh and Boyer−Ahmad (KB)
Kurdistan (KD)
Lorestan (LO)
Markazi (MK)
Mazandaran (MN)
North Khorasan (KS)
Qazvin (QZ)
Qom (QM)
Semnan (SM)
Sistan and Baluchistan (SB)
South Khorasan (KJ)
Tehran (TE)
West Azarbayejan (WA)
Yazd (YA)
Zanjan (ZA)

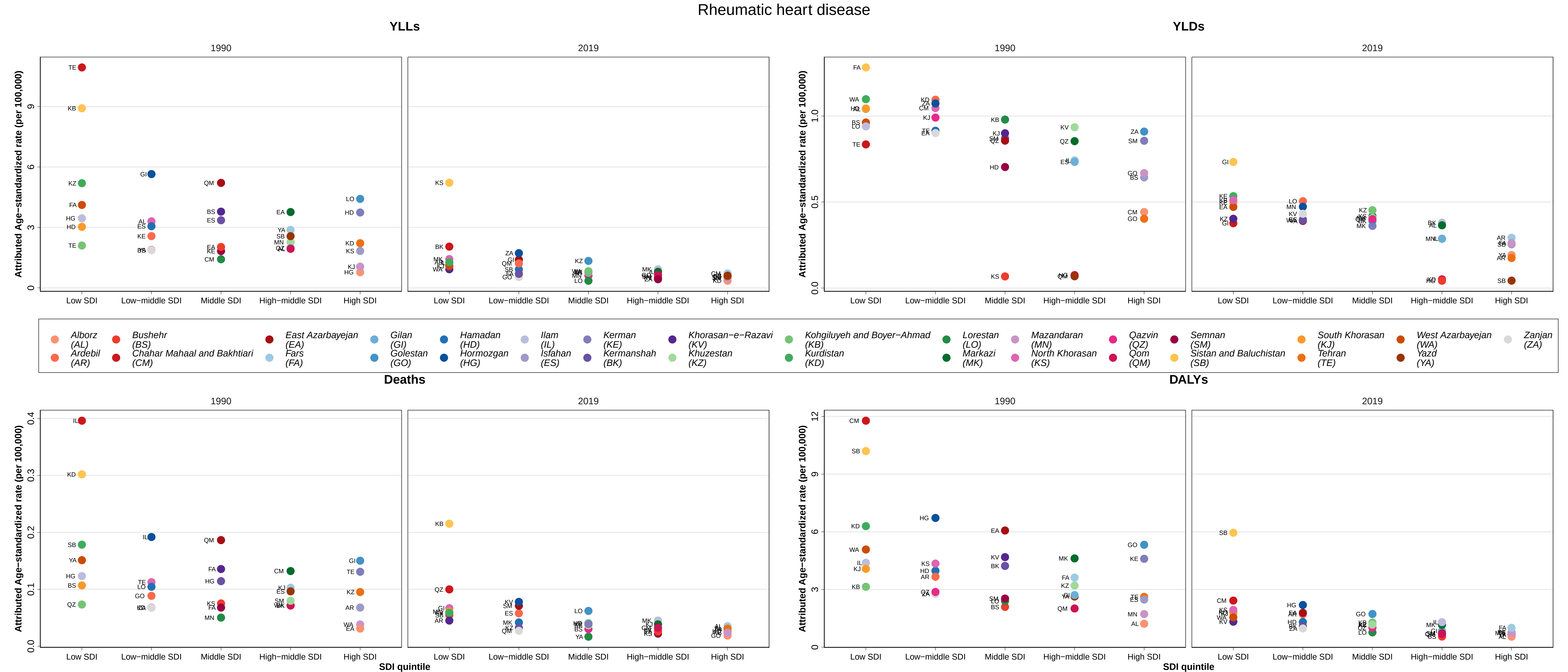
Rheumatic heart disease
YLLs
YLDs
Deaths
DALYs
1990
2019
Attributed Age−standardized rate (per 100,000)
Low SDI
Low−middle SDI
Middle SDI
High−middle SDI
High SDI
SDI quintile
Alborz (AL)
Ardebil (AR)
Bushehr (BS)
Chahar Mahaal and Bakhtiari (CM)
East Azarbayejan (EA)
Fars (FA)
Gilan (GI)
Golestan (GO)
Hamadan (HD)
Hormozgan (HG)
Ilam (IL)
Isfahan (ES)
Kerman (KE)
Kermanshah (BK)
Khorasan−e−Razavi (KV)
Khuzestan (KZ)
Kohgiluyeh and Boyer−Ahmad (KB)
Kurdistan (KD)
Lorestan (LO)
Markazi (MK)
Mazandaran (MN)
North Khorasan (KS)
Qazvin (QZ)
Qom (QM)
Semnan (SM)
Sistan and Baluchistan (SB)
South Khorasan (KJ)
Tehran (TE)
West Azarbayejan (WA)
Yazd (YA)
Zanjan (ZA)

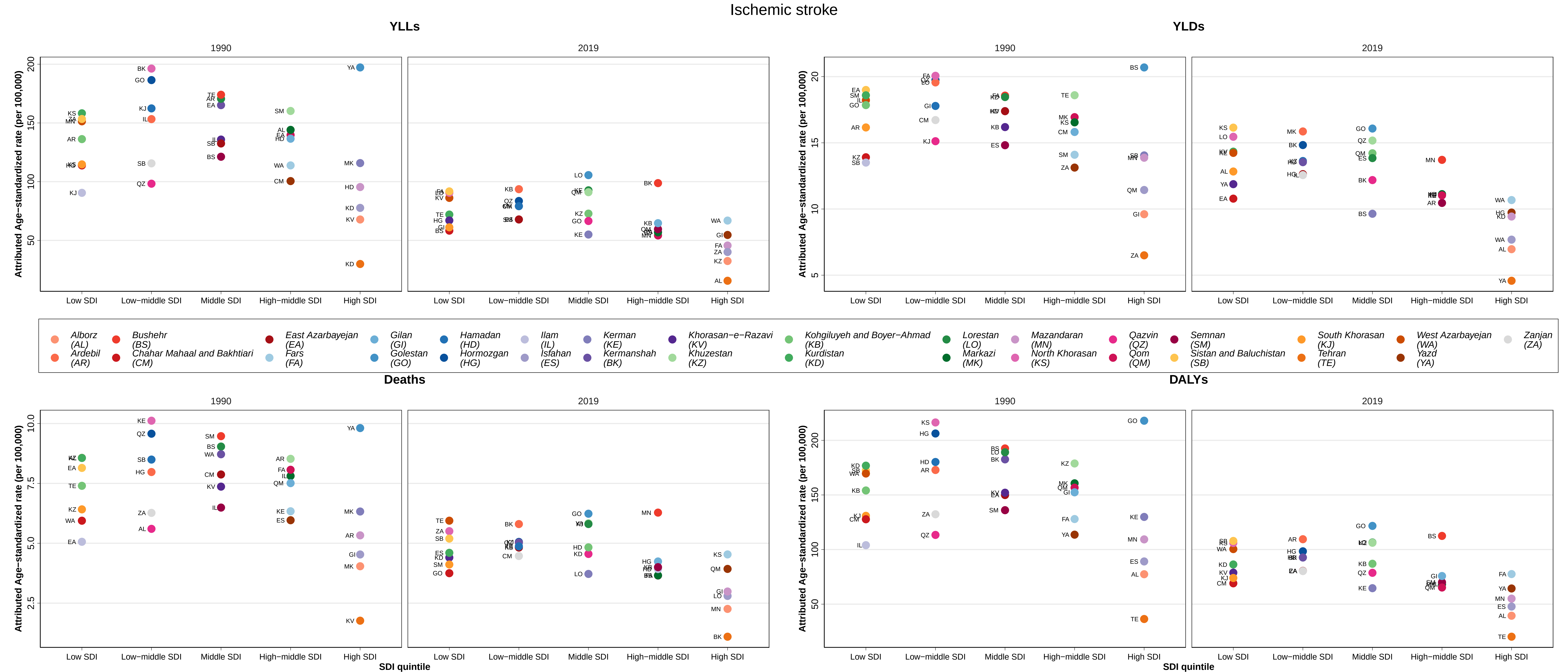
Ischemic stroke
YLLs
YLDs
Deaths
DALYs
1990
2019
Attributed Age−standardized rate (per 100,000)
Low SDI
Low−middle SDI
Middle SDI
High−middle SDI
High SDI
SDI quintile
Alborz (AL)
Ardebil (AR)
Bushehr (BS)
Chahar Mahaal and Bakhtiari (CM)
East Azarbayejan (EA)
Fars (FA)
Gilan (GI)
Golestan (GO)
Hamadan (HD)
Hormozgan (HG)
Ilam (IL)
Isfahan (ES)
Kerman (KE)
Kermanshah (BK)
Khorasan−e−Razavi (KV)
Khuzestan (KZ)
Kohgiluyeh and Boyer−Ahmad (KB)
Kurdistan (KD)
Lorestan (LO)
Markazi (MK)
Mazandaran (MN)
North Khorasan (KS)
Qazvin (QZ)
Qom (QM)
Semnan (SM)
Sistan and Baluchistan (SB)
South Khorasan (KJ)
Tehran (TE)
West Azarbayejan (WA)
Yazd (YA)
Zanjan (ZA)

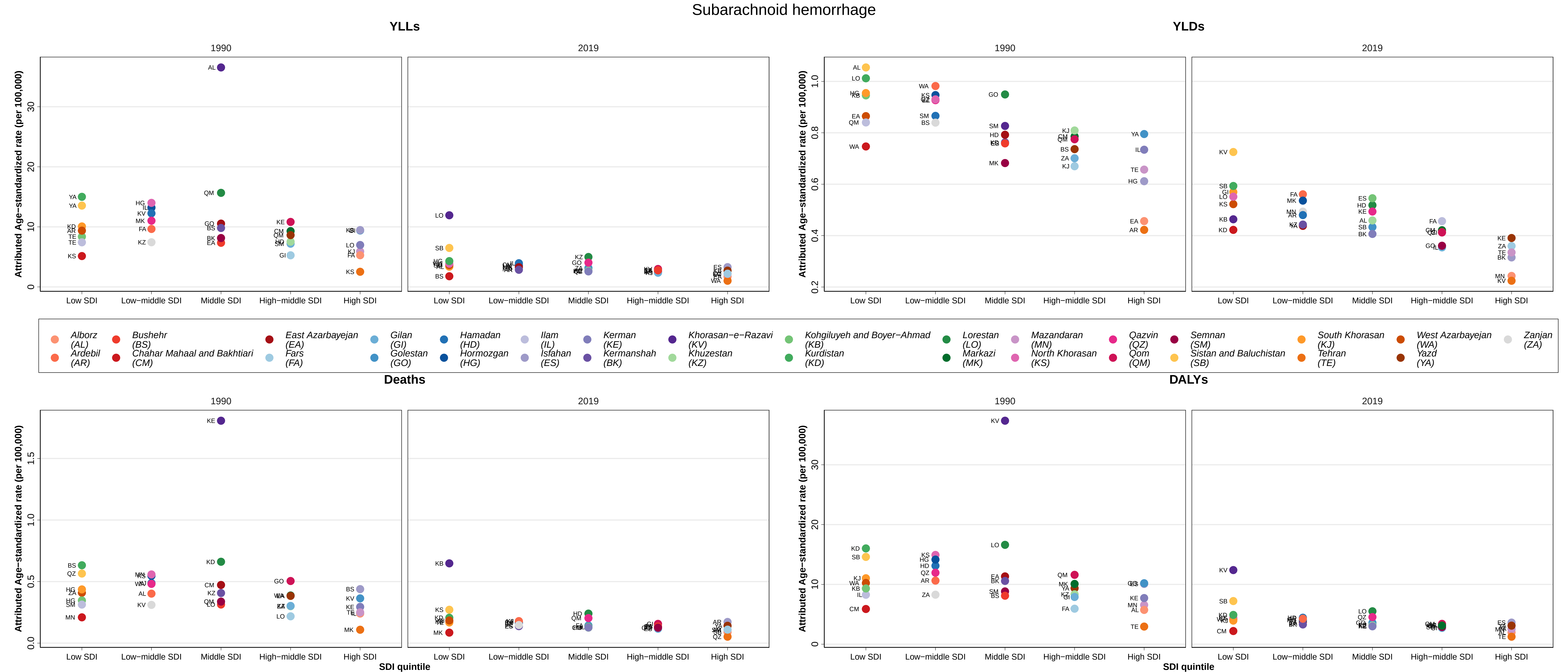

Subarachnoid hemorrhage
YLLs
YLDs
Deaths
DALYs
1990
2019
Attributed Age−standardized rate (per 100,000)
Low SDI
Low−middle SDI
Middle SDI
High−middle SDI
High SDI
SDI quintile
Alborz (AL)
Ardebil (AR)
Bushehr (BS)
Chahar Mahaal and Bakhtiari (CM)
East Azarbayejan (EA)
Fars (FA)
Gilan (GI)
Golestan (GO)
Hamadan (HD)
Hormozgan (HG)
Ilam (IL)
Isfahan (ES)
Kerman (KE)
Kermanshah (BK)
Khorasan−e−Razavi (KV)
Khuzestan (KZ)
Kohgiluyeh and Boyer−Ahmad (KB)
Kurdistan (KD)
Lorestan (LO)
Markazi (MK)
Mazandaran (MN)
North Khorasan (KS)
Qazvin (QZ)
Qom (QM)
Semnan (SM)
Sistan and Baluchistan (SB)
South Khorasan (KJ)
Tehran (TE)
West Azarbayejan (WA)
Yazd (YA)
Zanjan (ZA)

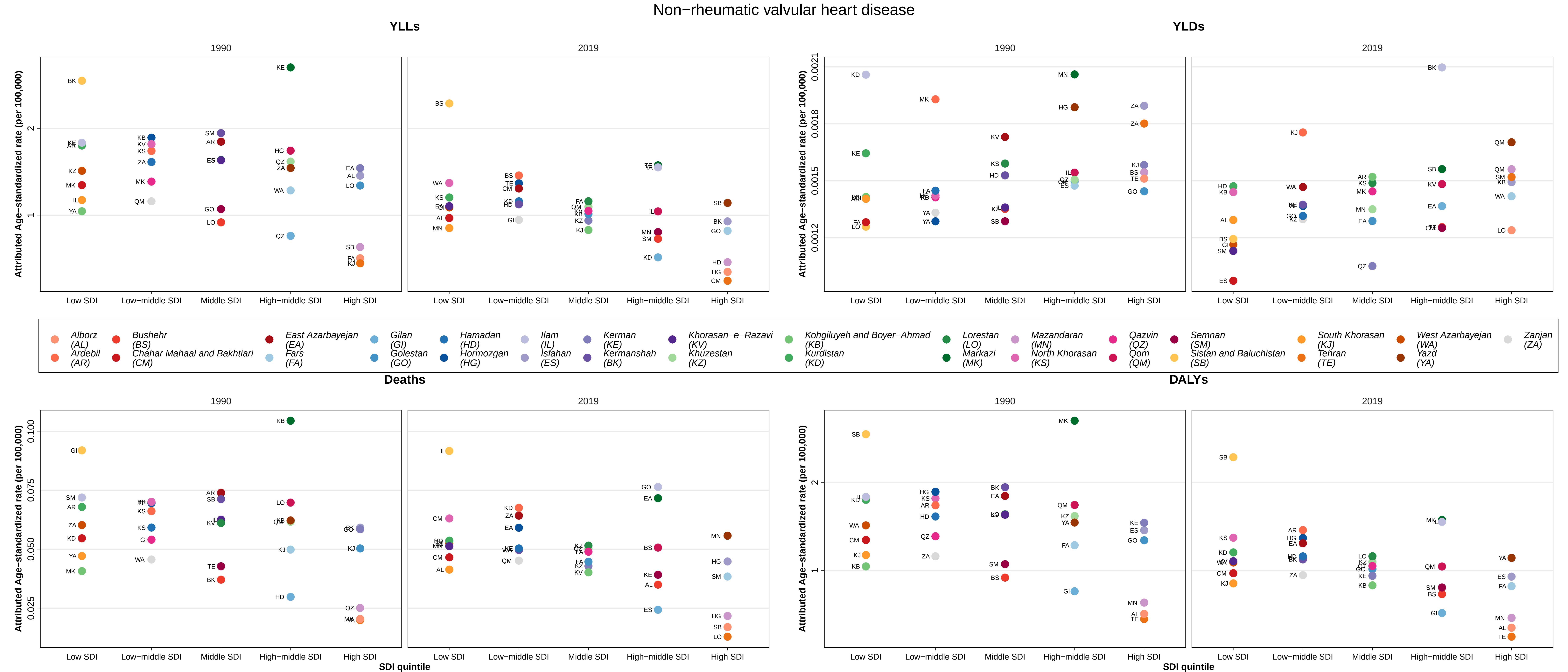

Non−rheumatic valvular heart disease
YLLs
YLDs
Deaths
DALYs
1990
2019
Attributed Age−standardized rate (per 100,000)
Low SDI
Low−middle SDI
Middle SDI
High−middle SDI
High SDI
SDI quintile
Alborz (AL)
Ardebil (AR)
Bushehr (BS)
Chahar Mahaal and Bakhtiari (CM)
East Azarbayejan (EA)
Fars (FA)
Gilan (GI)
Golestan (GO)
Hamadan (HD)
Hormozgan (HG)
Ilam (IL)
Isfahan (ES)
Kerman (KE)
Kermanshah (BK)
Khorasan−e−Razavi (KV)
Khuzestan (KZ)
Kohgiluyeh and Boyer−Ahmad (KB)
Kurdistan (KD)
Lorestan (LO)
Markazi (MK)
Mazandaran (MN)
North Khorasan (KS)
Qazvin (QZ)
Qom (QM)
Semnan (SM)
Sistan and Baluchistan (SB)
South Khorasan (KJ)
Tehran (TE)
West Azarbayejan (WA)
Yazd (YA)
Zanjan (ZA)

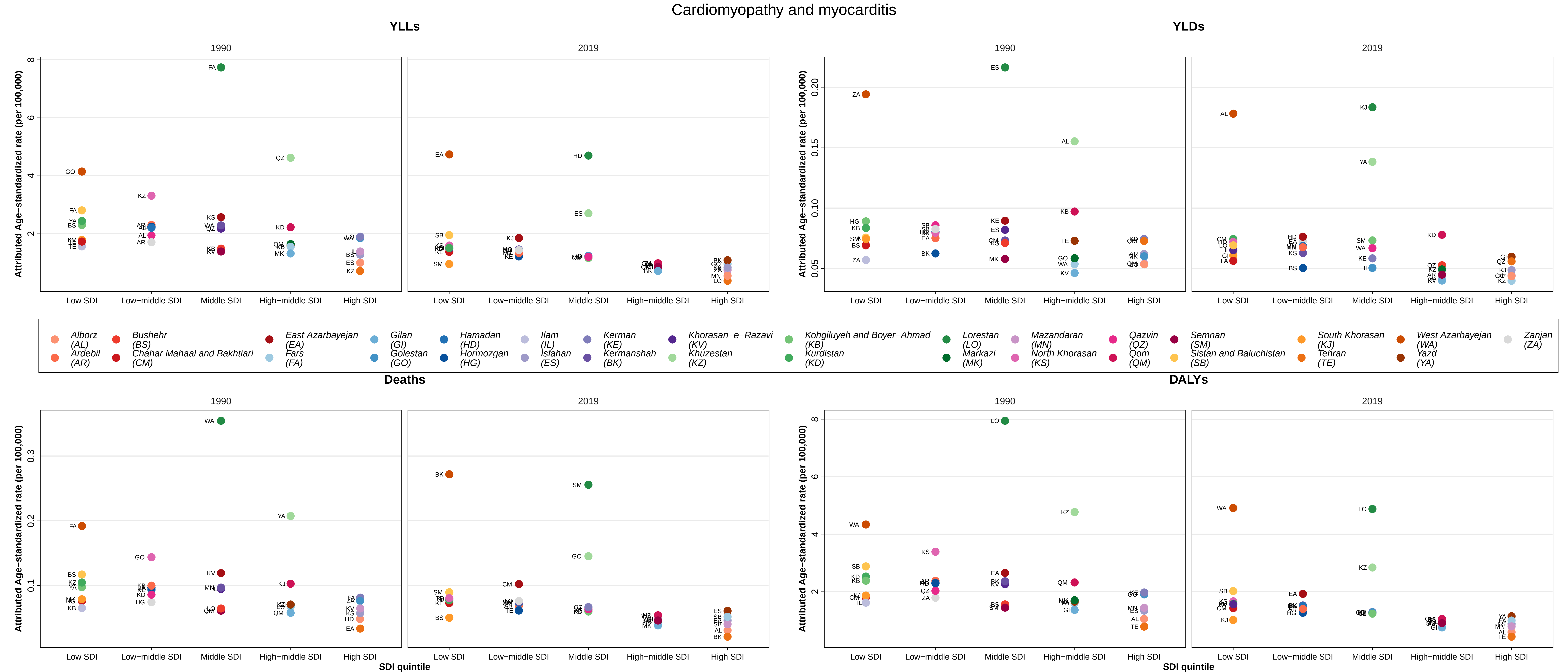

Cardiomyopathy and myocarditis
YLLs
YLDs
Deaths
DALYs
1990
2019
Attributed Age−standardized rate (per 100,000)
Low SDI
Low−middle SDI
Middle SDI
High−middle SDI
High SDI
SDI quintile
Alborz (AL)
Ardebil (AR)
Bushehr (BS)
Chahar Mahaal and Bakhtiari (CM)
East Azarbayejan (EA)
Fars (FA)
Gilan (GI)
Golestan (GO)
Hamadan (HD)
Hormozgan (HG)
Ilam (IL)
Isfahan (ES)
Kerman (KE)
Kermanshah (BK)
Khorasan−e−Razavi (KV)
Khuzestan (KZ)
Kohgiluyeh and Boyer−Ahmad (KB)
Kurdistan (KD)
Lorestan (LO)
Markazi (MK)
Mazandaran (MN)
North Khorasan (KS)
Qazvin (QZ)
Qom (QM)
Semnan (SM)
Sistan and Baluchistan (SB)
South Khorasan (KJ)
Tehran (TE)
West Azarbayejan (WA)
Yazd (YA)
Zanjan (ZA)
