# Supplementary material for: Unveiling the lead exposure attributed burden in Iran from 1990 to 2019 through the lens of the Global Burden of Disease study 2019
Source: Sci Rep. 2024 Apr 15;14:8688. doi: 10.1038/s41598-024-58823-z (PMC11018826; doi:10.1038/s41598-024-58823-z)
Supplement: Supplementary file 3 — Supplementary Information. [file 41598_2024_58823_MOESM3_ESM.docx]

**Supplementary file**

**The subnational burden of three Subtypes of CVD with the highest burden**

***IHD***

Subnationally, DALYs and YLLs rates almost halved from 1990 to 2019. In 2019, the lowest and the highest DALYs rate were 55.4 (27.6 to 90.6) and 396.5 (278.6 to 532.3), respectively and the lowest and highest rate of YLLs were 52.1 (26 to 84.8) and 387.6 (272.7 to 522.8), respectively, in the same provinces with minimum and maximum rates. Simultaneously, the ASDR spanned from 3.5 (1.8 to 5.6) to 22 (15 to 30.4). YLDs rate did not change significantly during these 30 years; in 2019 it was from 3.3 (1.5 to 5.9) to 9.2 (5.6 to 14.2).

***Stroke***

The rates of deaths, DALYs and YLLs nearly halved during these years among subnational provinces as follows: the death rate ranged from as low as 1.4 (0.7 to 2.1) to as high as 7.5 (5.1 to 10) in 2019. Simultaneously, the DALYs rate was 25.7 (12.6 to 40.9) to 166.5 (117.1 to 218), and the YLLs rate was from 20.4 (10.0 to 32.5) to 147.1 (102.0 to 194.7). Besides, the YLDs rate spanned from 5.3 (2.3 to 9.4) to 19.4 (12.3 to 27.4) in 2019.

***Hypertensive heart disease***

In terms of rate changes at the subnational level, the ASDR was estimated to span from 0.6 (0.1 to 1.5) to 10.9 (2.8 to 24.5) in 2019. Furthermore, the DALYs rate reached 8.8 (2.4 to 22.8) to 152.2 (51.5 to 321.1) in 2019. YLDs rate had an almost constant 30-year trend; in 2019, it spanned from as low as 1.5 (0.4 to 3.7) to as high as 7.7 (3.3 to 16.2) in 2019. However, the YLLs rate with a significant change reached 7.3 (1.9 to 19.8) to 146.3 (48.7 to 313) in 2019.

**CVDs subtypes’ burden that are not included in the manuscript**

***Endocarditis***

Nationally, the ASDR of endocarditis due to lead exposure decreased -25.8% (-45.7 to -2.2) from 1990 to 0 (0 to 0) in 2019. DALYs rate and YLLs rate declined almost equally from 1990 with -40.1% (-59.6 to -21.3) and -40.4% (-60.1 to -21.5) change respectively to the same rate of 0.5 (0.3 to 0.9) in 2019. YLDs rate showed the least change (-9.1% (-20.1 to 1.0)) to 0 (0 to 0) in 2019.

Subnationally, the ASDR spanned from 0.01 (Tehran) to 0.12 (North Khorasan) in 2019. In terms of DALYs, YLLs and YLDs, the ranges of rates in 2019 were as follows: DALYs range was from 0.1 (Tehran) to 2.7 (North Khorasan). Similarly, the YLLs rate had the same range and minimum and maximum provinces as the DALYs rate in 1990 and 2019. In addition, the YLDs rate range was 0 in all provinces in both years.

***Aortic aneurysm***

Nationally, the deaths rate decreased by -12.4% (-34.4 to 21.1) during the 30-year period to 0 (0 to 0.1) in 2019. The DALYs rate decreased by almost two times compared to the deaths rate with -26.4% (-45.8 to 2.2) change from 1990 and reached 0.9 (0.6 to 1.3) in 2019.

The deaths rate interval was almost constant over 30 years at the subnational level, and in 2019 was from 0.02 (Tehran) to 0.09 (Sistan and Baluchistan). At the same level and period, the DALYs rate range was 0.42 (Tehran) to 1.86 (Sistan and Baluchistan). Furthermore, the YLLs rate demonstrated a range of 0.4 (Tehran) to 1.9 (Sistan and Baluchistan).

***Stroke subtypes***

The burden attributed to all 3 stroke subtypes demonstrated declining patterns at the national and subnational provinces. Nationally, Subarachnoid hemorrhage accounted for an ASDR of 0.2(0.1 to 0.2) in 2019 with a -63.1% (-75.9 to -38.9) reduction over 30 years; simultaneously, a -66.2% (-77.3 to -47.0) reduction in DALYs rate to 3.7 (2.2 to 5.5) , a -67.6% (-78.6 to -47) reduction in YLLs to 3.3 (2.0 to 4.9) and a reduction of -45.6% (-59.0 to -34.9) to 0.4 (0.2 to 0.7) for YLDs were reported. The intracerebral hemorrhage showed a -57.3% (-64.8 to -47.6) reduction in deaths rate to 0.8 (0.5 to 1.1) in 2019. Furthermore, its DALYs, YLLs and YLDs diminished by -64.1% (-70.6 to -56.5), -65.1% (-71.8 to -57.5) and -44.4% (-54.3 to -35.8) during the course of the 30 years to 16.5 (10.6 to 23.1), 15.3 (9.8 to 21.4) and 1.2 (0.6 to 2) in 2019 respectively. Regarding the ischemic stroke, the deaths rate with -41.3% (-50.5 to -30.3), DALYs rate with -48.3% (-57.3 to -39.8), YLLs with -50.8% (-59.7 to -42.1) and YLDs with -28.3% (-36.0 to -21.9) diminution reached 3.7 (2.3 to 5.3), 67.2 (43.8 to 92.8), 56.8 (36.5 to 77.9) and 10.4 (5.9 to 16.0) in 2019, respectively.

***Cardiomyopathy and myocarditis***

all rates had a declining pattern over the 30- year time interval. Deaths rate with -25.8% (-48.5 to -2.0), DALYs with -36.7% (-55.1 to -17.5), YLLs with -37.5% (-56.3 to -17.7) and YLDs with -18.3% (-29.6 to -8.4) reduction from 1990 reached to 0.1 (0.0 to 0.1), 1.4 (0.8 to 2.0), 1.3 (0.8 to 1.9) and 0.1 (0.0 to 0.1) in 2019, respectively.

At the subnational level, the deaths rate spanned from 0.02 (Tehran) to 0.27 (West Azarbayejan) in 2019. Regarding the DALYs in 2019, rates related to 1990 at the lowest and highest levels were nearly halved, and the range was modified to 0.4 (Tehran) to 4.9 (West Azarbayejan). YLDs rate did not change during the mentioned period (0 to 0.2 (Lorestan)); however, the province with minimum rate changed from Gilan in 1990 to Fars in 2019. Moreover, the YLLs rate range was as low as 0.4 (Tehran) to as high as 4.7 (West Azarbayejan) in 2019.

***Non-rheumatic valvular heart disease***

Over 30 years, all rates had a declining pattern nationally. Deaths rate with -22.0% (-38.7 to 3.0), DALYs rate with -34.4% (-50.0 to -11.3), YLLs rate with -34.4% (-50.1 to -11.3) and YLDs with -12.0% (-24.2 to 0.7) reduction from 1990 reached 0.0 (0.0 to 0.1), 0.9 (0.5 to 1.3), 0.9 (0.5 to 1.3) and 0 (0 to 0) in 2019, respectively.

On the other hand, subnationally, the deaths rate spanned from as low as 0.01 (Tehran) to as high as 0.09 (Sistan and Baluchistan) in 2019. YLDs rates were almost zero in all provinces (by rounding to the first decimal place) in the same time interval. DALYs and YLLs had the same trend in their ranges in 1990 and 2019 (e.g., 0.2 (Tehran) to 2.3 (Sistan and Baluchistan) in 2019 was reported for both).

***Rheumatic heart disease***

The ASDR, DALYs, YLLs and YLDs rates revealed decreasing trends with a change of -62.4% (-78.7 to -44.0), -68.6% (-80.0 to -57.3), -71.6% (-83.1 to -59.2) and -56.4% (-75.1 to -41.9), respectively since 1990 to the following rates in 2019: 0 (0 to 0.1), 1.2 (0.6 to 2.1), 0.9 (0.5 to 1.6) and 0.3 (0.1 to 0.7).

The ASDR spanned from as low as 0.02 (Lorestan) to as high as 0.22 (Sistan and Baluchistan) at the subnational level in 2019. Regarding DALYs, YLLs and YLDs rates, an approximately 50% reduction in rates during the 30- year time interval was evident. In 2019 they reached 0.5 (Alborz) to 6.0 (Sistan and Baluchistan) for DALYs, 0.4 (Lorestan) to 5.2 (Sistan and Baluchistan) for YLLs and 0 (Bushehr) to 0.7 (Sistan and Baluchistan) for YLDs.

***Atrial fibrillation and flutter***

nationally the ASDR and YLLs rates demonstrated similar incremental patterns with a 30-year percent change of 20.1% (-5.5 to 73.3) to 0.1 (0.1 to 0.2) in 2019 for the deaths rate and a 7.3% (-14.3 to 48.8) increase to 1.7 (1.2 to 2.3) in 2019 for YLLs. Simultaneously, a declining pattern was evident for DALYs and YLDs rates, with a decrease of -0.9% (-12.3 to 15.1) to 3.7 (2.4 to 5.4) and -7.0% (-14.7 to -0.3) to 2.0 (1.1 to 3.2) in 2019 respectively.

At the subnational level, the deaths rate spanned from 0.06 (Tehran) to 0.24 (West Azarbayejan) in 2019. However, the YLDs range did not change so much during this period; it was from 1 to 3.4 and the YLLs rate was from 0.6 to 3.1 both for the same provinces as 1990 in 2019.

***Other cardiovascular and circulatory diseases***

DALYs, YLLs and YLDs revealed a -20.8% (-43.5 to -4.7), -17.4% (-44.5 to 1.5) and -35.5% (-48.6 to -25.2) change, respectively over 30 years to the following rates: 8.8 (5.9 to 11.9), 7.4 (5.1 to 9.9) and 1.4 (0.7 to 2.4) in 2019. On the other hand, ASDR had an increasing pattern with a 1.4% (-34.4 to 25.1) rise from 1990 to 0.4 (0.3 to 0.5) in 2019.

At the subnational level, regarding the deaths rate, unlike the national level, there was a reduction in the rate range; however, it was not significant. In 2019 it reached 0.05 (Tehran) to 1.23 (Ardebil). The provinces with minimum and maximum rates were the same and constant for all rates (Tehran with minimum deaths, DALYs, YLDs and YLLs rates and Ardebil with maximum rates) during the mentioned time interval. In 2019, the DALYs rate spanned from 1.2 to 26.4, YLDs from 0.4 to 3.9 and YLLs from 0.7 to 22.5.

**Points to consider regarding the CVD’s burden trends**

Most CVDs subtypes demonstrated a declining pattern in their attributed burden in the 30-year period. However, the following subtypes were exceptions: peripheral artery disease which showed this pattern at both national and subnational levels. Nationally, the age-standardized deaths, DALYs and YLLs rates showed an increase of 225.7% (89.3 to 379.8), 35.1% (11.2 to 77.4) and 206.8% (86.9 to 345.1) from 1990 to 0 (0 to 0), 0.3 (0.2 to 0.5) and 0.1 (0.1 to 0.2) in 2019, respectively. However, the YLDs rate demonstrated a different decreasing pattern.

Concurrently, subnational provinces revealed an increase in the ASDR from 1990, which reached 0 to 0.05 in 2019. DALYs rate reached 0.1 to 0.9 in 2019. Similarly, the range of YLLs rate increased during these years and spanned from 0 to 0.7 in 2019. However, the YLDs rate had a stable pattern during this time interval and was constantly from 0.1 to 0.3. Moreover, atrial fibrillation and flutter showed incremental patterns for ASDR and YLLs rate at national level (with the change of 20.1% (-5.5 to 73.3) to 0.1 (0.1 to 0.2) for the deaths rate and a 7.3% (-14.3 to 48.8) increase to 1.7 (1.2 to 2.3) in 2019 for YLLs. Besides, the ASDR attributed to other cardiovascular and circulatory diseases increased by 1.4% (-34.4 to 25.1) to 0.4 (0.3 to 0.5) in 2019.
